# RAB5c orchestrates LC3-associated phagocytosis to promote microbicidal function of macrophages

**DOI:** 10.1126/sciadv.adz0196

**Published:** 2026-05-08

**Authors:** Edismauro Garcia Freitas-Filho, Isabella Zaidan, Daniel Leonardo Alzamora-Terrel, Carolina Bifano, Marlon Fortes-Rocha, Patrícia Alves de Castro, Renan Eugênio Araujo Piraine, Camila Figueiredo Pinzan, Caroline Patini de Rezende, Emilio Boada-Romero, Thomas Wileman, Fausto Almeida, Gustavo Henrique Goldman, Oliver Florey, Larissa Dias Cunha

**Affiliations:** ^1^ Departamento de Biologia Celular e Molecular e Bioagentes Patogênicos, Faculdade de Medicina de Ribeirão Preto (FMRP), Universidade de São Paulo (USP), Ribeirão Preto, SP, Brazil.; ^2^ Departamento de Ciências Farmacêuticas, Faculdade de Ciências Farmacêuticas de Ribeirão Preto, USP, Ribeirão Preto, Brazil.; ^3^ Departamento de Bioquímica e Imunologia, FMRP, USP, Ribeirão Preto, SP, Brazil.; ^4^ Department of Immunology, St. Jude Children’s Research Hospital, Memphis, TN, USA.; ^5^ Norwich Medical School, University of East Anglia, Norwich, Norfolk, UK.; ^6^ Signalling Programme, Babraham Institute, Cambridge, UK.

## Abstract

Noncanonical conjugation of ATG8 proteins, including LC3, to single membranes implicates the autophagy machinery in cell functions unrelated to metabolic stress. One such pathway is LC3-associated phagocytosis (LAP), which aids in phagosome maturation and subsequent signaling upon cargo uptake mediated by certain innate immunity-associated receptors. Here, we show that a specific isoform of RAB5 GTPases, the molecular switches controlling early endosome traffic, is necessary for LAP. We demonstrate that RAB5c regulates phagosome recruitment and function of complexes required for phosphatidylinositol 3-phosphate [PI(3)P] and reactive oxygen species (ROS) generation by macrophages. RAB5c facilitates phagosome translocation of the V-ATPase transmembrane core, which is needed for ATG16L1 binding and consequent LC3 conjugation. RAB5c depletion impaired macrophage elimination of the fungal pathogen *Aspergillus fumigatus* and disruption of the V-ATPase–ATG16L1 axis increased susceptibility in vivo. Thus, early endosome-to-phagosome trafficking can be selectively engaged to promote pathogen elimination by directing phagosomal maturation toward LAP.

## Teaser

Early endosome trafficking can be selectively modulated to enable LC3 lipidation on phagosomes.

## INTRODUCTION

Phagocytosis of invading microorganisms and dead cells plays critical roles in development, homeostasis, infection resistance, and immunoregulation. The processes of phagosome formation and maturation are tightly controlled to ensure efficient cargo digestion and to initiate signaling cascades that modulate phagocyte function (*1*). Consequently, defects in cargo recognition, engulfment, or degradation of phagosome content can lead to pathogenic outcomes.

Macroautophagy, hereafter referred to as autophagy, is a degradative pathway in which the formation of an autophagolysosomal compartment facilitates the clearance of damaged organelles, nutrient recycling, and removal of cytosolic pathogens (*2*). Phagocytosis initiated by activation of transmembrane pattern recognition receptors (such as Toll-like receptors TLR2 and TLR4 and C-type lectin receptor Dectin-1), immunoglobulin receptor FcγR, and receptors for dying cells, triggers a noncanonical autophagy pathway known as LC3-associated phagocytosis (LAP) (*3*–*6*). In contrast to autophagy, LAP uses components of the autophagy machinery to couple receptor activation with phagosome maturation, shaping phagocyte effector functions and its consequences upon gene expression (*7*). Deficiencies in LAP impair the clearance of diverse pathogens by macrophages (*8*), including *Aspergillus fumigatus* (*9*–*11*), *Salmonella enterica* (*12*), and *Listeria monocytogenes* (*13*–*15*). The microbicidal activity of interferon-γ (IFN-γ) against *Toxoplasma gondii* also relies on LAP (*16*). Moreover, LAP fine-tunes antigen processing and presentation by controlling phagolysosome maturation in dendritic cells (*6*, *17*, *18*). Upon the uptake of cell corpses, LAP in myeloid cells sustains an immunosuppressive microenvironment that promotes tumor tolerance (*4*).

LAP and autophagy are evolutionarily conserved yet distinct pathways. The ULK1/2-ATG13 complex that initiates autophagy is dispensable for LAP (*19*). Instead, LAP requires the assembly of a unique Beclin-1–VPS34 class III phosphatidylinositol 3-kinase [PI(3)K] complex containing Rubicon (RUBCN) to generate phosphatidylinositol 3-phosphate [PI(3)P] on the phagosome membrane (*5*, *20*). During LAP, but not autophagy, ionic imbalance caused by reactive oxygen species (ROS) production via reduced form of nicotinamide adenine dinucleotide phosphate (NADPH) oxidase 2 (NOX2) complex triggers the assembly of phagosome proton-pumping V–adenosine triphosphatase (V-ATPase) (*21*). Assembled V-ATPase serves as a tether for the E3 ligase-like ATG16L1-ATG5-ATG12 complex on the phagosome (*21*–*23*), independently of autophagy components FIP200 and WIPI2b (*24*), and promotes the covalent conjugation of ATG8 family proteins [microtubule-associated protein 1 light chain 3 (MAP1LC3, referred as LC3) A/B/B2/C; GABARAP/L1/L2] to phosphatidylethanolamine and phosphatidylserine (PS) residues of the phagosome membrane during LAP (*24*–*26*).

Phagosomes are dynamic organelles that undergo continuous remodeling during maturation through sequential fusion with endosomes (*27*). Membrane recognition and fusion require homotypic interaction between Ras-associated binding guanosine-triphosphatases (RAB-GTPases). Although RAB GTPase-mediated targeting and fusion of early endosomes (EEs) and late endosomes (LEs) with maturing phagosomes is well-documented (*28*–*31*), the significance of intercompartment communication for LAP is unknown. Meanwhile, despite the identification of several components of LAP, how they are recruited to the phagosome and integrated into a unified signaling pathway remains poorly understood. Elucidating the spatiotemporal organization of LAP could provide critical insights for therapeutically targeting innate immune responses in the context of infections, cancer, and immune dysregulation.

Here, we investigated the importance of EE-phagosome interactions during LAP. We found that RAB5c, a specific isoform of EE GTPase RAB5, is required for LC3 lipidation on phagosomes. We show that RAB5c regulates the localization and activity of the class III PI(3)K complex and controls the traffic of the transmembrane core of the V-ATPase to the phagosome. We further reveal that the V-ATPase binding domain of ATG16L1 is required for resistance to infection in mice. This study establishes a differential role for RAB5c in controlling phagosome maturation during LAP and demonstrates that phagosome ROS exerts microbicidal activity against *A. fumigatus* by acting as a second messenger for V-ATPase assembly and phagosome maturation.

## RESULTS

### RAB5c depletion impairs LAP

**Fig. 1.**
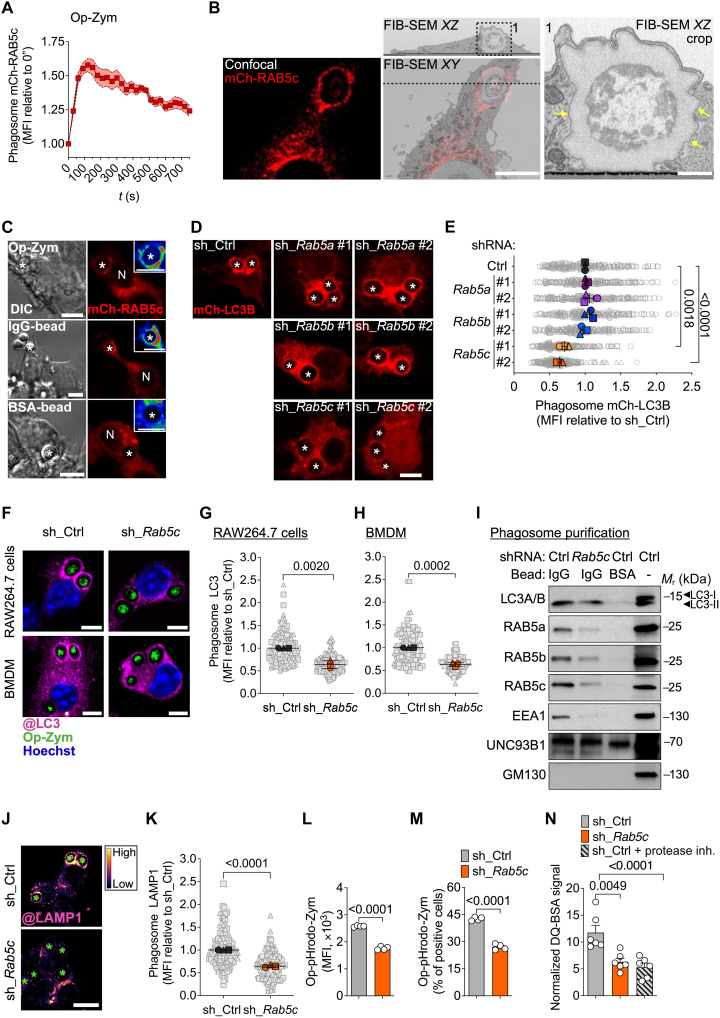
RAB5c depletion impairs LAP. (**A**) mCh-RAB5c MFI at phagosomes (*n* = 12) of RAW264.7 cells fed Op-zym (see movie S1). (**B**) CLEM analysis of Op-zym^+^ phagosome in RAW264.7-mCh-RAB5c cells. Left: Fluorescence image. Middle: overlay (bottom) and corresponding TEM cross section (top). Right: Magnified inset; arrows indicate single membrane. Scale bars, 5 μm (left and middle) and 1 μm (right). (**C**) Confocal images of RAW264.7-mCh-RAB5c cells stimulated with Op-zym, IgG-beads, or BSA-beads for 5 min. Insets fluorescence intensity (show as RAINBOW LUT). Scale bar, 5 μm. DIC, differential interference contrast. (**D** and **E**) Confocal images (D) and mCh-LC3B MFI at phagosomes (E) for RAW246.7 cells expressing scrambled shRNA (sh_Ctrl) or indicated shRNAs (#1; #2) fed Op-zym. Scale bar, 5 μm. (**F** to **H**) Confocal images (F) and MFI of LC3 at phagosomes for RAW264.7 cells (G) and BMDMs (H) expressing sh_Ctrl or sh_*Rab5c* fed Op-zym. Scale bars, 5 μm. (**I**) Immunoblot of LC3A/B, RAB5a/b/c, EEA1, UNC93B1 (loading control), and GM130 (purification control) in IgG-bead– or BSA-bead–containing phagosomes purified from RAW264.7 cells expressing sh_Ctrl or sh_*Rab5c*. *M*_r_, relative molecular mass. (**J** and **K**) Confocal images (shown as FIRE LUT) (J) and LAMP1 MFI at phagosomes (K) for RAW264.7 cells fed Op-zym. Scale bar, 10 μm. (**L** and **M**) MFI (L) and percentage (M) of Op-pHrodo-zym in RAW264.7 cells analyzed by flow cytometry. (**N**) MFI of opsonized DQ-BSA–coated beads in RAW264.7 cells. Colored symbols [(A), (E), (G), (H), and (K)] or bars [(L) to (N)] represent means of biological replicates, each indicated as a gray (phagosome) or white (sample) object. Shaded area (A) or error bars, ±SEM. Statistical comparison between groups are unpaired Student’s *t* test. Data are pooled from two (A) or three [(E), (G), (H), and (K)] independent experiments or are representative of four (I), three [(L) and (M)], or two (N) independent experiments. *, phagosomes; N, nuclei.

In mammalian cells, three paralogs of RAB5 GTPase—*Rab5a*, *Rab5b*, and *Rab5c*—are associated with the control of EE trafficking (*32*). However, studies that distinguish the activities of individual RAB5 genes, particularly those describing nonredundant functional roles among them, are scarce (*33*–*36*). Of note, the isoform RAB5c associates with phagosomes containing *Mycobacterium tuberculosis* (*37*), *S. enterica* serovar Typhimurium (*38*), and *A. fumigatus* (*39*) and is required for the maturation of the phagosome containing these pathogens. Pathogenic *Legionella pneumophila* actively avoids fusion of RAB5c-containing EE with its vacuole through the activity of the bacterial effector VipD, arresting vacuole maturation and ultimately blocking lysosomal fusion (*40*–*42*). This suggests that targeting RAB5c is a pathogen-driven subversion strategy to impair phagolysosome biogenesis, thereby allowing the establishment of a replicative niche. Real-time analysis of RAW264.7 macrophages expressing a mCherry-tagged murine RAB5c isoform (mCherry-RAB5c) showed the recruitment of mCherry-RAB5c to the vicinity of phagosomes containing opsonized zymozan (Op-zym), a stimulus previously shown to induce LAP (*21*). RAB5c recruitment occurred early upon Op-zym uptake and lasted only during the first minutes of phagosome trafficking (movie S1, fig. S1A, and Fig. 1A). The specific association of mCherry-RAB5c with the single lipid bilayer of the phagosome was independently confirmed by correlative light–electron microscopy (CLEM) (Fig. 1B). We also detected mCherry-RAB5c localized around phagosomes within the first 5 min of engulfment in macrophages stimulated with immunoglobulin G (IgG)–coupled beads (IgG-beads) to induce LAP, but not in macrophages fed bovine serum albumin–coupled beads (BSA-beads), which do not induce LAP and serve as an inert stimulus (*25*, *26*). Our data indicate trafficking of RAB5c to early phagosomes during LAP (Fig. 1C).

To probe the role of early endomembrane trafficking regulated by RAB5 during LAP, we performed gene silencing using two different short hairpin RNA (shRNA) constructs for each *Rab5* paralog in RAW264.7 cells expressing fluorescent murine LC3B (mCherry-LC3B). We found that depletion of RAB5c, but not RAB5a or RAB5b, reduced the presence of mCherry-LC3B on phagosomes containing Op-zym (Fig. 1, D and E). We validated commercially available antibodies against each murine RAB5 isoform and confirmed silencing specificity (fig. S1, B and C). Real-time analysis confirmed that knockdown of RAB5c (*Rab5c* KD) impaired the conjugation of mCherry-LC3B to phagosomes instead of causing its removal (movie S2 and fig. S1D). Immunofluorescence confocal microscopy confirmed less endogenous LC3 decorating phagosomes containing Op-zym in *Rab5c* KD RAW264.7 cells, without affecting particle uptake (Fig. 1, F and G, and fig. S1E). Similarly, *Rab5c* silencing in bone marrow–derived macrophages (BMDMs) also reduced LC3 lipidation to phagosomes (Fig. 1, F and H; and fig. S1, F and G). Additionally, depletion of RAB5c by lentiviral CRISPR-Cas9 in RAW264.7 cells precluded LC3 recruitment to Op-zym–containing phagosomes (fig. S1, H to L), without affecting phagocytosis (fig. S1M). Dominant-negative (S35N) or constitutively active (Q80L) mutations, which impair guanosine diphosphate–guanosine 5′-triphosphate (GTP) exchange and GTP hydrolysis, respectively, prevented the increase in LC3 lipidation induced by RAB5c overexpression, indicating that RAB5c GTPase activity is required for LAP (fig. S1N).

Next, we performed phagosome purification in control and *Rab5c* KD RAW264.7 macrophages. LC3-II was enriched in phagosomes containing IgG-beads compared with BSA-beads (Fig. 1I and fig. SO). Consistent with our microscopy observations, we found a reduction in LC3 levels in IgG-bead–containing phagosomes from *Rab5c* KD macrophages (Fig. 1I and fig. S1O). Notably, IgG-bead–containing phagosomes from control macrophages were enriched in all RAB5 isoforms and the RAB5 effector early endosome antigen 1 (EEA1), a tethering factor that mediates endosomal vesicle docking and fusion with target membranes (*43*), compared with those containing BSA-beads (Fig. 1I and fig. S1, P to S). Conversely, *Rab5c* KD reduced EEA1, RAB5a, and RAB5b levels in the phagosomes with IgG-beads, consistent with the idea that RAB5c facilitates EE recruitment to the nascent phagosomes during LAP (Fig. 1I and fig. S1, P to S).

Last, we determined that *Rab5c* silencing reduced the levels of LAMP-1 in phagosomes containing Op-zym, indicating that the fusion of LEs/lysosomes with phagosomes is impaired (Fig. 1, J and K). We next evaluated LAP-regulated phagosome acidification using Op-zym labeled with the pH-sensitive probe pHrodo Red by real-time imaging and flow cytometry and found reduced phagosome acidification in *Rab5c* KD macrophages (Fig. 1, L and M; and fig. S1, T and U). Consistently, phagolysosome proteolytic degradation was diminished in *Rab5c* KD macrophages, as assessed by microscopy of beads coated with dye-quenched BSA (DQ-BSA), a self-quenched fluorescent protease substrate (Fig. 1N and fig. S1V). These results confirm that RAB5c is required for phagolysosome maturation during LAP. Collectively, these findings support that RAB5c regulates EE-phagosome communication during LAP and is required for LC3 conjugation and subsequent phagolysosome maturation.

### RAB5c depletion impairs PI(3)P production and stable assembly of NOX2 machinery on phagosomes but still increases phagosome ROS production

**Fig. 2.**
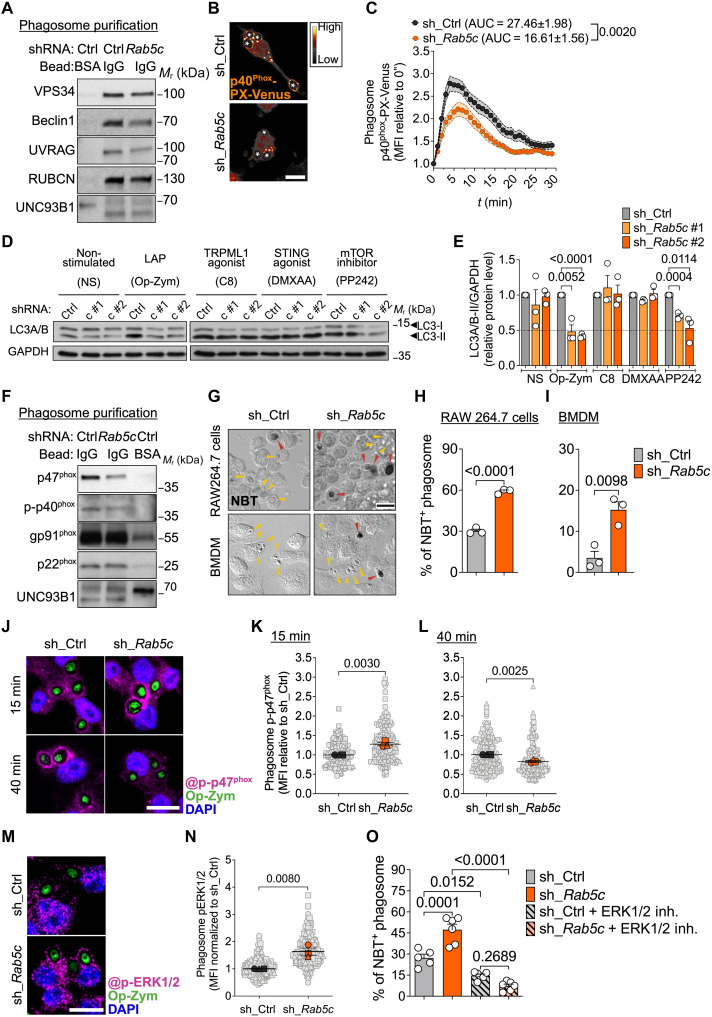
RAB5c depletion impairs PI(3)P production and stable assembly of NOX2 machinery on phagosomes but increases phagosome ROS production. RAW264.7 cells or BMDMs expressing sh_Ctrl or sh_*Rab5c*. (**A**) Immunoblot of class III PI(3)K complex in BSA-bead– or IgG-bead–containing phagosomes from RAW264.7 cells. (**B** and **C**) Confocal images (shown as SMART LUT) (B) and p40^phox^-PX-Venus MFI at phagosomes (*n* = 18) (C) in RAW264.7-p40^phox^-PX-Venus cells fed Op-zym. *, phagosomes (see movie S3). (**D** and **E**) Immunoblot (D) and quantification (E) of LC3A/B-II in RAW246.7 cells ± Op-zym (25 min), C8 (2 μM; 25 min), DMXAA (50 μg/ml; 1 hour), and PP242 (1 μM; 2 hours). Glyceraldehyde-3-phosphate dehydrogenase (GAPDH), loading control. NS, nonstimulated. (**F**) Immunoblot of NOX2 components in IgG-bead– or BSA-bead–containing phagosomes from RAW264.7 cells. (**G** to **I**) NBT assay in macrophages fed Op-zym. DIC images (G) and percentage of NBT^+^ phagosomes in RAW264.7 cells (H) and BMDMs (I). Arrowheads, NBT^+^ (red) or NBT^−^ (yellow). (**J** to **L**) Confocal images (J) and p-p47^phox^ MFI at phagosomes in RAW264.7 cells fed Op-zym for 15 (K) or 40 (L) min. (**M** and **N**) Confocal images (M) and MFI (N) of p-ERK1/2 in RAW264.7 cells fed Op-zym (15 min). (**O**) Percentage of NBT^+^ phagosomes in RAW264.7 cells ± ERK1/2 inhibitor (100 μM) for 10 min before Op-zym. Colored symbols [(C), (K), (L), and (N)] or bars [(E), (H), (I), and (O)] represent means of biological replicates, each indicated as a gray (phagosome) or white (sample) object. Shaded area (C) or error bars, ±SEM. Statistical comparisons are unpaired Student’s *t* test [(C), (E), (H), (I), (K), (L), and (N)] or analysis of variance (ANOVA) and Tukey’s multiple comparisons (O). Data are pooled from two (C) or three [(E), (K), (L), and (N)] independent experiments or are representative of three to five [(A) and (F)], two [(B), (I), and (O)], or three [(D), (G), and (H)] independent experiments. Scale bars, 10 μm.

Next, we examined the role of RAB5c in the assembly of the LAP machinery onto the phagosome. First, we assessed the role of RAB5c on the recruitment and function of UV radiation resistance associated (UVRAG)–Beclin-1–VPS34–VPS15 class III PI(3)K complex during LAP. We found a reduction in VPS34, Beclin-1, and UVRAG levels on the phagosome membrane of IgG-beads purified from *Rab5c*-silenced macrophages (Fig. 2A and fig. S2, A to C). RUBCN, which directly binds VPS34 and is also required for PI(3)P generation on the phagosome membrane (*5*, *20*), was also reduced on IgG-bead–containing phagosomes from *Rab5c* KD macrophages (Fig. 2A and fig. S2D).

VPS34 is a RAB5 effector (*44*), and RAB5a recruits and induces VPS34 kinase activity in EEs (*45*). To test the role of RAB5c in the function of VPS34 during LAP, we evaluate PI(3)P levels by imaging phagosomal association of a genetically encoded probe based on the PI(3)P-binding domain of p40^phox^ (p40^phox^-PX-Venus) (*46*). We found by real-time confocal analysis that the binding of p40^phox^-PX-Venus to phagosomes containing Op-zym was reduced upon silencing of *Rab5c* (Fig. 2, B and C, and movie S3)*.* These findings corroborate that RAB5c helps recruit RUBCN and the class III PI3K complex onto phagosomes and is required for local PI(3)P generation during LAP.

The class III PI(3)K complex containing UVRAG–Beclin-1–VPS34–VPS15 promotes autophagosome maturation by directing autophagosome fusion with LEs (*47*), a function that does not require RUBCN (*47*–*49*). Of note, we found that RAB5c depletion reduced autophagosome formation in response to macroautophagy induction with mechanistic target of rapamycin (mTOR) inhibitor PP242 (*50*), as assessed by immunoblot analysis of LC3-I to LC3-II conversion and the immunostaining of endogenous LC3 puncta by confocal microscopy (Fig. 2, D and E, and fig. S2E). Endolysosomal pH perturbations caused by multiple stimuli—such as viroporins, ionophores, lysosomotropic drugs, activation of endolysosomal channel TRPML1 (transient receptor potential cation channel, mucolipin subfamily, member 1), and STING (stimulator of interferon gene protein) activation—can also induce the noncanonical conjugation of LC3 to endolysosomal single membranes (*21*, *25*). In these cases, lipidation occurs independently of macroscale endocytic engulfment and RUBCN-VPS34 function (*25*). RAB5c depletion did not affect LC3-II levels in response to TRPML1 activation by the agonist C8 or activation of STING by 5,6-dimethylxanthenone-4-acetic acid (DMXAA) (Fig. 2, D and E, and fig. S2E). These data further support that RAB5c is required for VPS34 kinase activity to induce lipidation of LC3 during LAP and is not universally required for noncanonical autophagy.

RUBCN and VPS34 contribute to the activity of the NOX2 complex in the phagosome membrane through the binding of the transmembrane NOX2 p22^phox^ subunit to RUBCN (*5*, *20*, *51*) and the cytosolic p40^phox^ subunit to PI(3)P, generated by VPS34 (*52*, *53*). NOX2 generates ROS within the phagosome lumen, establishing a RUBCN/PI(3)P/ROS axis essential for LAP progression. We therefore examined the presence of NOX2 complex components on phagosomes in the absence of RAB5c. IgG-bead–containing phagosomes purified from *Rab5c* KD macrophages had reduced levels of cytosolic subunits p47^phox^ and phosphorylated p40^phox^ but retained similar levels of the transmembrane subunits p22^phox^ and gp91^phox^ when compared with phagosomes purified from control cells (Fig. 2F and fig. S2, F to I).

Next, we assessed phagosome ROS generation by measuring the deposition of insoluble formazan using the nitroblue tetrazolium (NBT) assay. Unexpectedly, we found an increased deposition of insoluble formazan mediated by O_2_^−^ in Op-zym–containing phagosomes from *Rab5c* KD RAW264.7 cells and BMDMs in comparison with those from control cells (Fig. 2, G to I). We obtained similar results by measuring luminol oxidation during Op-zym phagocytosis in the presence of horseradish peroxidase (HRP) (fig. S2J). The elevated ROS burst in *Rab5c* KD macrophages in response to Op-zym was suppressed by the NOX2 inhibitor diphenyleneiodonium (DPI) at nanomolar concentrations (fig. S2K) and was not associated with increased levels of mitochondrial ROS (fig. S2L), indicating that the higher ROS production remained NOX2-dependent. RAB5c depletion also increased ROS production in response to IgG-beads (fig. S2M), but not in response to phorbol 12-myristate 13-acetate (PMA) (fig. S2N), which induces protein kinase C–dependent NOX2 activation at the plasma membrane (*54*). These results support that RAB5c modulates NOX2-mediated ROS production during LAP.

To understand how depletion of RAB5c could increase ROS levels despite reduced NOX2 complex assembly at the phagosome membrane, we imaged the translocation of cytosolic p47^phox^, an early carrier of the cytosolic p47^phox^-p67^phox^-p40^phox^ ternary complex to p22^phox^-containing phagosomes (*55*, *56*), at different time points using confocal microscopy. In *Rab5c* KD macrophages, we found an early peak of p47^phox^ recruitment 15 min after Op-zym internalization compared with control cells (fig. S2, O and P). However, this increase was not sustained over time, with p47^phox^ levels declining at 40 min poststimulation, consistent with our observations in purified phagosomes (Fig. 2F). Similarly, phagosomes from RAB5c-depleted macrophages exhibited a transient peak of decoration of p-p47^phox^, a phosphorylation event required for its binding to membrane-spanning p22^phox^ in murine cells (Fig. 2, J to L) (*55*). In human monocytes infected with *A. fumigatus*, active extracellular signal–regulated kinase 1/2 (ERK1/2) mitogen-activated protein kinase traffics to phagosomes and controls p47^phox^ localization and phosphorylation, which is essential for ROS production during LAP (*9*). Thus, we tested whether ERK1/2 contributes to elevated ROS production in Op-zym–containing phagosomes of *Rab5c* KD macrophages. First, *Rab5c* silencing increased phosphorylated ERK1/2 (p-ERK1/2) phagosomal decoration (Fig. 2, M and N). Second, pharmacological inhibition of ERK catalytic activity with Ulixertinib (BVD-523) (*57*) abolished the enhanced ROS production, as assessed by the NBT assay (Fig. 2O). We also observed increased ERK-dependent ROS levels in phagosomes of *Rab5c* KD macrophages fed opsonized red blood cells (fig. S2, Q and R), indicating that RAB5c regulates ROS production during LAP in response to distinct cargo. Collectively, these data suggest that RAB5c depletion hinders the stable assembly of NOX2 on the phagosome membrane. Nevertheless, the loss of RAB5c paradoxically increases phagosome ROS production by promoting ERK1/2 accumulation on phagosomes, which correlates with premature p47^phox^ activity.

### RAB5c controls the assembly of the V-ATPase in the phagosome during LAP

**Fig. 3.**
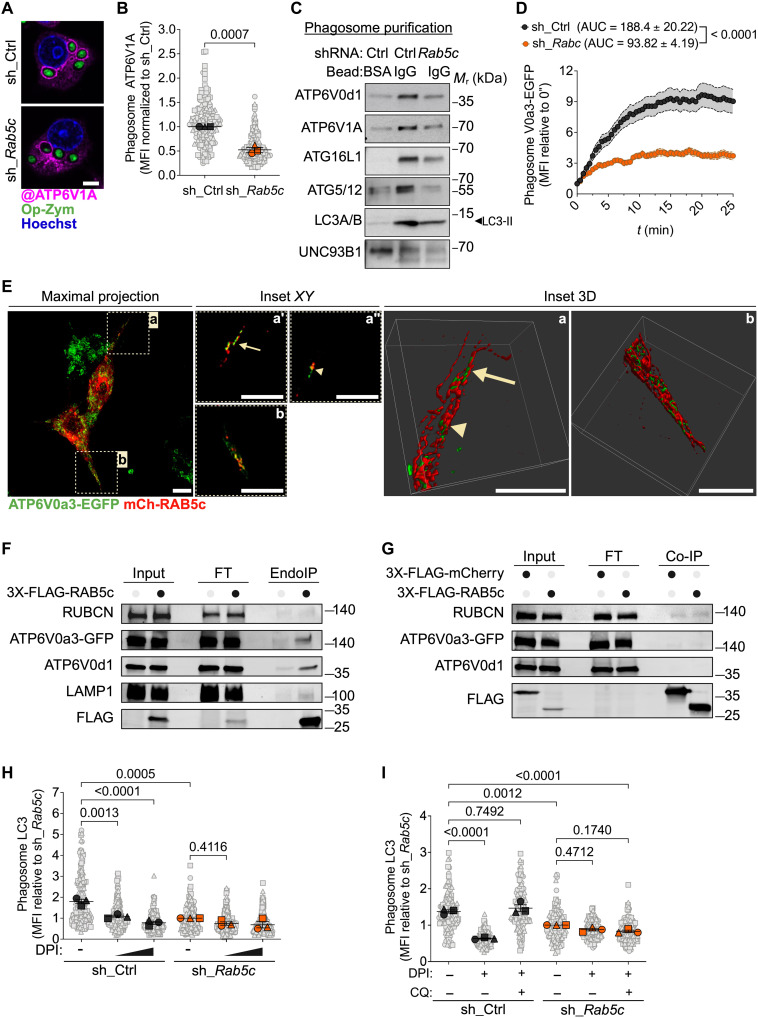
RAB5c controls the assembly of the V-ATPase in the phagosome during LAP. RAW264.7 cells expressing sh_Ctrl or sh_*Rab5c*. (**A** and **B**) Confocal images (A) and MFI (B) of ATP6V1A at phagosomes in cells fed Op-zym. (**C**) Immunoblot of components of the V-ATPase complex and ATG8 lipidation machinery in purified BSA-bead– or IgG-bead–containing phagosomes. (**D**) ATP6V0a3-EGFP MFI at phagosomes (*n* = 20) of cells fed Op-zym (see movie S4). (**E**) Enhanced-resolution confocal image of cells expressing ATP6V0a3-EGFP and mCh-RAB5c. Left: Maximal-intensity projection. Middle: Single *Z*-sections of insets a and b. Right: Three-dimensional projections of insets a and b. (**F**) Immunoblot of endosome immunoprecipitation (EndoIP) from RAW264.7-3X-FLAG-RAB5c cells. Loaded fractions correspond to 1.3% of input and flow-through (FT) and 20% of immunoprecipitation (IP) eluate. (**G**) Immunoblot of coimmunoprecipitation (co-IP) from RAW264.7 cells overexpressing 3X-FLAG-mCherry or 3X-FLAG-RAB5c. Loaded fractions correspond to 2% of input and FT and 20% of IP eluate. Blots were probed with the indicated antibodies. (**H**) LC3 MFI at phagosomes upon treatment with DPI (0.61 or 5 μM) before Op-zym. (**I**) LC3 MFI at phagosomes upon treatment with DPI (5 μM) or DPI and chloroquine (CQ; 100 μM) before Op-zym. Colored symbols [(B), (D), (H), and (I)] represent means of biological replicates (phagosome), each indicated as a gray object. Shaded area (D) or error bars, ±SEM. Statistical comparison between groups are unpaired Student’s *t* test [(B) and (D)] or ANOVA and Tukey’s multiple comparisons [(H) and (I)]. Data are pooled from three [(B), (H), and (I)] or two (D) independent experiments or are representative of three [(A) and (F)], four to five (C), or two [(E) and (G)] independent experiments. Scale bars, 5 μm.

Because depletion of RAB5c did not cause a reduction of NOX2-mediated ROS production, we asked whether impairment of LC3 lipidation in *Rab5c* KD macrophages was due to regulation of other downstream events in the LAP cascade. During LAP, ROS-induced ionic imbalance by proton (H^+^) consumption and consequent pH raise triggers the binding of cytosolic V1 to the transmembrane V0 component of the phagosome V-ATPase (*21*). Once the complex is assembled, ATG16L1 binds to ATP6V1H, a subunit of the V1 component of the V-ATPase, through its tryptophan–aspartic acid (WD) repeat-containing C-terminal domain (*22*, *23*). This interaction enables the docking of the ATG12-ATG5-ATG16L1 complex onto single membranes and directs the site of LC3 lipidation (*21*–*23*). Confocal microscopy analysis revealed a reduction in the engagement of ATP6V1A (a V1 subunit) on Op-zym–containing phagosomes in *Rab5c* KD macrophages compared with control cells (Fig. 3, A and B). Of note, the depletion of RAB5c did not affect the expression of ATP6V1A (fig. S3, A and B). ATP6V1A levels were also reduced in IgG-bead–containing phagosomes purified from *Rab5c* KD macrophages (Fig. 3C and fig. S3C). Last, *Rab5c* silencing diminished the recruitment of ATG16L1 and ATG5/12 on purified phagosomes, thus confirming that RAB5c is required for recruiting core ATG proteins that orchestrate the conjugation of LC3 to phospholipids (Fig. 3C and fig. S3, D and E).

We found that RAB5c depletion decreased the levels of the V0 subunit ATP6V0d1 in purified IgG-beads, indicating that RAB5c controls the traffic of the V-ATPase transmembrane core to the phagosome (Fig. 3C and fig. S3F). Live confocal imaging of RAW264.7 cells expressing an enhanced green fluorescent protein (EGFP)–tagged V0 subunit (ATP6V0a3-EGFP) fed Op-zym revealed that V0 acquisition by phagosomes occurred upon contact between V0^+^ organelles and phagosome membrane (movie S4). However, this transfer of V0 to phagosomes was disrupted upon *Rab5c* silencing (movie S4 and Fig. 3D). Enhanced-resolution confocal imaging of RAW264.7 cells coexpressing ATP6V0a3-EGFP and mCherry-RAB5c revealed that the V0 transmembrane component and RAB5c can be found in close proximity yet remain spatially segregated, on a subset of tubular and vesicular structures (Fig. 3E). To address whether V0 core localizes to RAB5c^+^ compartments, we purified EE by affinity capture using endosome immunoprecipitation (EndoIP) (*58*) and using 3X-FLAG-RAB5c as bait. EE purified by RAB5c immunoprecipitation contained ATP6V0a3-EGFP and ATP6V0d1 (Fig. 3F). Of note, these RAB5c^+^ endosomes were enriched for LAMP1, which has been reported on EE (*58*, *59*), but did not contain detectable RUBCN (Fig. 3F). Last, we did not detect a stable or potentially direct association between RAB5c and these V-ATPase subunits in parallel coimmunoprecipitation (co-IP) assays (Fig. 3G), indicating that V0 core is unlikely to function as a RAB5c effector and is instead delivered to phagosomes during LAP via EE trafficking.

Our data, so far, could explain failed LC3 lipidation on phagosomes in *Rab5c*-deficient macrophages despite the presence of the triggering ROS signal. Alternatively, aberrant ROS production in *Rab5c* KD macrophages could disrupt the reversible assembly of V-ATPase on phagosomes. To test the latter hypothesis, we treated Op-zym–stimulated *Rab5c* KD macrophages with low-dose DPI (61 nM). Although this treatment reduced ROS levels to those found in control cells (fig. S2K), it failed to restore ATP6V1A recruitment to the phagosome membrane in *Rab5c* KD cells (fig. S3G). Consistently, lowering ROS levels in *Rab5c* KD macrophages did not rescue LC3 lipidation (Fig. 3H). Last, forced sequestration of luminal H^+^ with chloroquine, which rescues ATP6V1A recruitment to phagosomes upon treatment with high-dose DPI (5 μM) (*21*), restored LC3 conjugation in control, but not in RAB5c KD macrophages (Fig. 3I). Thus, defective V-ATPase assembly upon RAB5c depletion and consequent disruption of the LC3 conjugation cascade are not directly cause by excessive ROS. Collectively, these findings support the notion that RAB5c participates in LAP by controlling the recruitment and activity of class III PI3K and the trafficking of the transmembrane V0 core of the V-ATPase to the phagosome.

### RAB5c-mediated V-ATPase assembly confers macrophage resistance to *A. fumigatus*

Generation of phagosomal ROS by NOX2 contributes for the elimination of *A. fumigatus* by macrophages (*9*, *10*, *60*). Additionally, the fungal wall dihydroxy naphthalene (DHN)–melanin of *A. fumigatus* is a pathogenicity mechanism that excludes p22^phox^ of the phagosome and blocks LC3 lipidation (*10*), further suggesting that LAP activation is required for fungal killing and protection against invasive infection.

**Fig. 4.**
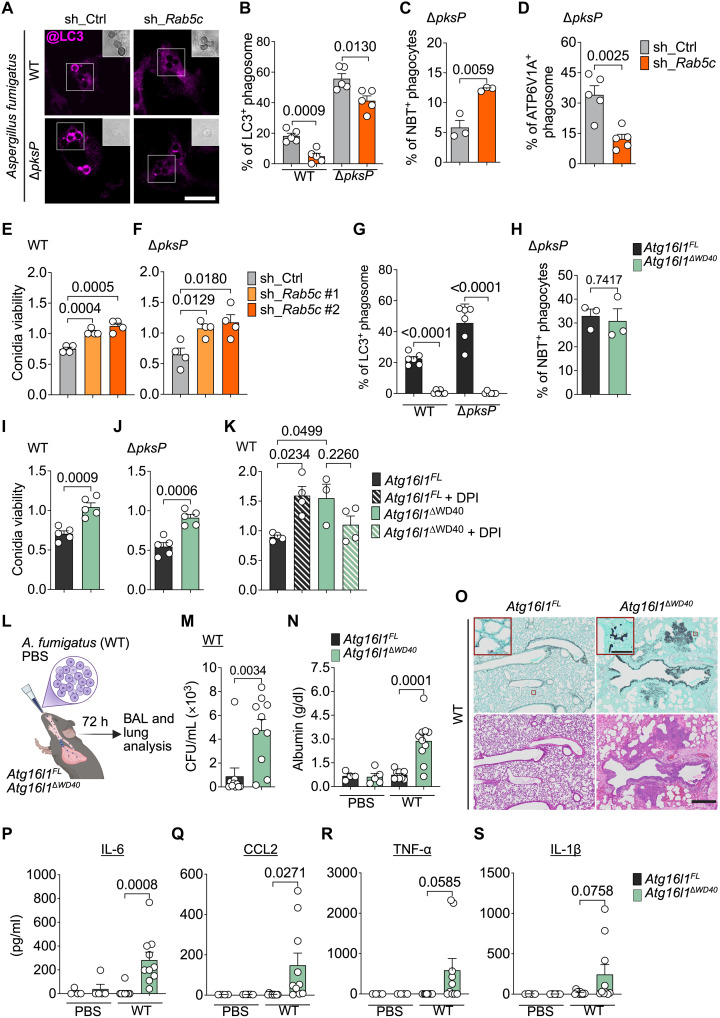
RAB5c-mediated V-ATPase assembly confers macrophage resistance to *A. fumigatus.* (**A** to **F**) BMDMs expressing sh_Ctrl or sh_*Rab5c* were stimulated with wild-type (WT; ATCC46645) or Δ*pksP A. fumigatus* conidia. [(A) and (B)] Confocal images (A) and percentage (B) of LC3^+^ phagosomes. Insets: DIC of conidia. Scale bar, 10 μm. (C) Percentage of NBT^+^ phagocytes. (D) Percentage of ATP6V1A^+^ phagosomes after 1 hour. [(E) and (F)] Viability of WT (E) and Δ*pksP* (F) conidia. (**G** and **H**) *Atg16l1^FL^* and *Atg16l1*^Δ*WD40*^ BMDMs were stimulated with WT or Δ*pksP* conidia. Percentage of LC3^+^ phagosomes (G) and NBT^+^ phagocytes (H). (**I** to **K**) Viability of WT conidia (I), Δ*pksP* conidia (J), and WT conidia ± DPI (5 μM) (K) in *Atg16l1^FL^* and *Atg16l1*^Δ*WD40*^ BMDMs. (**L**) Schematics of *A. fumigatus* lung infection. Created in BioRender. L. Cunha (2026), https://BioRender.com/l4k0vfu. h, hours. (**M**) Fungal loads in the bronchoalveolar lavage fluid (BAL) samples of infected mice. (**N**) Quantification of albumin in BAL. (**O**) Bright-field images of lung sections stained with Grocott-Gomori’s methenamine silver (top) or hematoxylin and eosin (bottom). Scale bar, 500 μm. Insets: *A. fumigatus* hyphae. Scale bar, 50 μm. (**P** to **S**) Quantification of interleukin-6 (IL-6) (P), CCL2 (Q), tumor necrosis factor–α (TNF-α) (R), and IL-1β (S) in BAL. Bars [(B) to (K), (M), (N), and (P) to (S)] represent means of biological replicates (sample or mice), each indicated as a white object. Error bars, ±SEM. Statistical comparison between groups are unpaired Student’s *t* test [(B) to (J), (M), (N), and (P) to (S)] or ANOVA and Tukey’s multiple comparisons (K) between indicated groups. Data are representative of two [(A) to (D), (G), (H), (J), (K), and (M) to (S)] or three [(E), (F), and (I)] independent experiments.

To investigate the importance of RAB5c and LAP for macrophage function, we first assessed their role in LAP induction upon engulfment of *A. fumigatus* conidia by BMDMs using microscopy analysis*.* Silencing of *Rab5c* decreased LC3 conjugation to phagosomes containing the *A. fumigatus* wild-type (WT) strain [American Type Culture Collection (ATCC), 466645] (Fig. 4, A and B). *Rab5c* silencing also impaired LC3 recruitment to phagosomes with the pigmentless melanin-deficient Δ*pksP* isogenic mutant, which induces LAP more efficiently (Fig. 4, A and B) (*9*–*11*). Using stimulation with Δ*pksP* conidia, we observed that reduced LC3 lipidation occurred despite increased ROS production in the phagosomes of *Rab5c* KD BMDMs (Fig. 4C and fig. S4A), mirroring the effect of LAP induction with Op-zym (Fig. 2, G and I). Of note, the increase in luminal ROS was associated with more recruitment of p-p47^phox^ and ERK1/2 to the phagosomes of *Rab5c* KD BMDMs (fig. S4, B and C), thus corroborating that depletion of RAB5c disrupts normal ERK1/2 traffic and NOX2 function during LAP. Still and consistent with our previous data (Fig. 3, A and B), depletion of RAB5c resulted in less engagement of ATP6V1A onto phagosomes containing Δ*pksP* conidia (Fig. 4D and fig. S4D), despite the increased ROS production.

Next, we addressed the importance of RAB5c for macrophage killing of *A. fumigatus.* We found that *Rab5c* silencing caused defective conidial elimination of WT (Fig. 4E) and Δ*pksP* (Fig. 4F) strains in comparison with control BMDMs, without affecting conidium uptake (fig. S4, E and F). Notably, conidium uptake and killing of Δ*pksP* was not impaired by silencing *Rab5a* or *Rab5b* (fig. S4, G to I). Collectively, our data support that RAB5c regulates LAP induction upon engulfment of *A. fumigatus* and is required for efficient clearance of the pathogen by macrophages.

Because RAB5c promotes V-ATPase assembly in the phagosome membranes, we sought to determine the mechanistic link between RAB5c, V-ATPase, and ATG16L1 to the resistance against *A. fumigatus*. To that end, we used an in vitro infection model with BMDMs from mice homozygous for two premature stop codons in *Atg16l1*, which prevent translation of the C-terminal WD40 domain (*Atg16l1*^Δ*WD40*^) (*61*). ATG16L1 lacking the WD40 domain cannot bind to V-ATPase assembled on single membranes and, therefore, fails to engage noncanonical LC3 to lipidation on single-membrane compartments while leaving macroautophagy intact (*24*, *61*). We found that conjugation of LC3 to phagosomes containing WT or Δ*pksP* conidia was severely impaired in *Atg16l1*^Δ*WD40*^ BMDM (Fig. 4G). However, the expression of ATG16L1^ΔWD40^ did not affect phagosome ROS production in response to infection, in agreement with NOX2 activation occurring earlier in the LAP cascade (Fig. 4H) (*20*, *21*). *Atg16l1*^Δ*WD40*^ BMDMs internalized conidia similar to *Atg16l1^FL^* control cells (fig. S4, J and K) but eliminated WT and Δ*pksP* conidia less efficiently (Fig. 4, I and J). Further, we found that treatment with DPI impaired the clearance of *A. fumigatus* in *Atg16l1^FL^*, but not in *Atg16l1*^Δ*WD40*^, BMDMs, supporting that ROS generation by NOX2 requires V-ATPase and ATG16L1 axis to exert its microbicidal function (Fig. 4K). Similar to the expression of *Atg16l1*^Δ*WD40*^ alleles, ablation of *Rubcn* did not affect the engulfment but impaired the killing of WT conidia (fig. S4, L and M), thus confirming the role of RUBCN in the control of *A. fumigatus* infection by macrophages.

Last, we assessed the role of the V-ATPase–ATG16L1 axis in a nonlethal model of lung infection of immunocompetent mice with WT *A. fumigatus* conidia (Fig. 4L). Expression of ATG16L1^ΔWD40^ resulted in a higher recovery of viable conidia from the bronchoalveolar lavage fluid, suggesting impaired in vivo clearance of the pathogen by pulmonary phagocytes (Fig. 4M). Additionally, elevated albumin levels, indicative of lung damage, were detected in the lavage fluid of *Atg16l1*^Δ*WD40*^, but not *Atg16l1^FL^*, mice, relative to their uninfected littermates (Fig. 4N). Histopathological examination revealed the presence of *A. fumigatus* hyphae within the lung parenchyma of *Atg16l1*^Δ*WD40*^ mice 72 hours postinfection (Fig. 4O and fig. S4, N and O). Further, lungs from infected *Atg16l1*^Δ*WD40*^ mice presented clear signs of higher immune cell infiltration compared with *Atg16l1^FL^* mice (Fig. 4O). We also found increased levels of proinflammatory cytokines interleukin-6 (IL-6), C-C motif chemokine ligand 2 (CCL2), and tumor necrosis factor–α (TNF-α) and variable levels of IL-1β in the lavage fluid of infected *Atg16l1*^Δ*WD40*^ mice (Fig. 4, P to S). Collectively, these data support the conclusion that disruption of the ATPase-ATG16L1 interaction results in impaired resistance and exacerbated immunopathology in response to *A. fumigatus*. Together, our findings establish that LAP is required for efficient killing of *A. fumigatus* by macrophages and that noncanonical autophagy promotes immunity against infection in vivo*.*

## DISCUSSION

While RAB5 GTPases, markers of EE interactions, are frequently observed during the maturation of phagosomes formed upon recognition and internalization of pathogenic cargo, their functional role in this process remains poorly defined. Notably, RAB5 isoforms are often assumed to be functionally interchangeable, leaving gaps in our understanding of isoform-specific roles. Our findings demonstrate that RAB5c plays a nonredundant role in the LAP pathway during phagosome biogenesis, facilitating the efficient elimination of fungal pathogens. Specifically, we show that RAB5c (i) orchestrates the transport and function of components responsible of PI(3)P generation, an early event of the LAP cascade; (ii) modulates the levels of ROS, a key second messenger; and (iii) enables LC3 lipidation by mediating the trafficking of the V-ATPase transmembrane core to the phagosome. By coordinating these processes, RAB5c ensures the spatiotemporal organization of the molecular platforms required for LAP signal transduction.

At steady state, the RUBCN–UVRAG–Beclin-1 complex localizes to EEA1-positive endosomes independently of PI(3)P (*49*, *62*–*64*). In this context, our data suggest that RAB5c may facilitate the transport of RUBCN-containing endosomes to phagosomes. However, whether RAB5c influences PI(3)P production by recruiting RUBCN or by directly binding to VPS34 on phagosomes remains to be determined. Notably, we found that *Rab5c* silencing reduces LC3 lipidation during autophagy, a process that does not require RUBCN (*2*, *48*, *49*). Therefore, RAB5c may independently regulate both the localization of RUBCN and its binding partners to phagosomes, as well as site-specific activation of VPS34.

Receptors triggering LAP share a common signature of PS clustering around a basic KRKPKK motif in their C-terminal domain, to which RUBCN can directly bind on the nascent phagosomes (*5*). Only VPS34 that interacts with RUBCN can produce PI(3)P (*5*). Therefore, it is possible that the assembly of a functional complex comprising RAB5c-VPS34-RUBCN-PS is required to stabilize PI(3)K CIII at the phagosome membrane once all components are recruited. Furthermore, it would be interesting to investigate whether PS clustering serves as the initial signal for RAB5c recruitment to phagosomes, given that K-Ras and RAC1 localization and activation are preferentially directed toward PS-rich membranes (*65*, *66*).

The binding of p40^phox^ to PI(3)P is required for accumulation and stable retention of the ternary cytosolic NOX2 components at phagosomes (*55*). In the absence of RAB5c, reduced PI(3)P generation may destabilize the NOX2 complex, potentially explaining the observed decrease in activated p40^phox^ and p47^phox^ in purified phagosomes following LAP induction. Notably, Ueyama *et al.* (*52*, *55*) previously demonstrated that FcγR-mediated ROS production in macrophages depends on p40^phox^ PI(3)P binding only when p47^phox^ is partially phosphorylated at sites enabling its interaction with p22^phox^. Thus, NOX2 activity and premature ROS burst in RAB5c*-*depleted macrophages may occur independently of prolonged NOX2 complex stability. In the absence of RAB5c on phagosomes, sustained ERK1/2 activity might drive oxidase activation, favored by p47^phox^. Because we confirmed previous findings that p-ERK1/2 localizes to phagosomes and is required for ROS production during *A. fumigatus* infection (*9*), we speculate that RAB5c and EE interactions are necessary for the removal of p-ERK1/2 during phagosome maturation. This process could fine-tune ROS production as excessive luminal ROS levels have been shown to accelerate V-ATPase assembly and LC3 lipidation on phagosomes while slowing acidification in monocytic cells (*21*).

Our study demonstrates that RAB5c is required for the localization of V0 component of the V-ATPase to the phagosome, which subsequently enables V1 engagement in response to ROS. Our findings suggest that V0 recruitment is a regulated process dependent on communication between compartments of the endomembrane system. This idea aligns with previous biochemical evidence showing enrichment of V0 subunits in EE (*58*, *67*). Most recently, mapping of endosome composition in human cells by EndoIP coupled to cross-linking mass spectrometry identified spatial proximity between RAB5c and ATP6V0a1, but not RAB5a or RAB5b, on the same EE, consistent with a transient or membrane-facilitated interaction (*59*). In our system, microscopy and EE purification similarly place V0 within RAB5c^+^ compartments. Yet, we could not detect a direct association by co-IP, arguing against V0 as a canonical RAB5c effector. ATP6V0a3 transfer to nascent phagosomes can occur via tubular structures extending from lysosomes (*68*), suggesting that phagosome-lysosome interactions may occur early in the maturation process. Further supporting this model, during LAP, the lysosomal marker LAMP1 localizes to phagosomes before LC3 lipidation (*21*). Given that RAB5c can be found adjacent to ATP6V0a3 on a subset of tubular structures, that RAB5c-containing EE is LAMP1^+^, and that RAB5c is required for transfer of V0 to phagosomes, it is possible that RAB5c, directly or indirectly, facilitates these interactions. Additional studies will be necessary to determine how RAB5c-regulated EE trafficking governs the route delivering V0 component to phagosomes.

The discovery that ROS-dependent conjugation of LC3 to the phagosome membrane occurs in response to *A. fumigatus* infection led to the hypothesis that LAP, rather than a direct microbicidal mechanism, underlies the critical role of NOX2 and phagosomal ROS in macrophage resistance to the pathogen. Substantial data supporting this hypothesis comes from studies in murine models and human myeloid cells. However, the genetic basis for this connection has primarily relied on hematopoietic-specific ablation of *Atg5* or *Atg7*, genes shared by both LAP and autophagy, which renders macrophages and mice susceptible to infection (*9*, *10*, *69*). The opposing effects of RAB5c on phagosomal ROS production and LC3 lipidation, by regulating different machineries that participate in LAP, allowed us to dissect the role of ROS in macrophage-mediated pathogen killing*.* Our findings demonstrate that NOX2-derived ROS eliminates *A. fumigatus* by triggering the V-ATPase–ATG16L1 axis of LAP. Macrophages deficient in *Rab5c* exhibited impaired V-ATPase assembly and LC3 lipidation, rendering them more susceptible to infection despite the elevated ROS levels. To provide definitive genetic evidence, we used a mutant mouse strain with disrupted V-ATPase–ATG16L1 interaction (*61*), selectively impairing noncanonical autophagy. This model conclusively demonstrated that LAP is essential for macrophage-mediated *A. fumigatus* elimination. While our study establishes noncanonical autophagy as a critical mechanism for host resistance in vivo, future research is needed to determine the contributions of specific cell types to the susceptibility associated with *Atg16l1*^Δ*WD40*^, as has been demonstrated for infection with influenza A (*61*).

## MATERIALS AND METHODS

### Reagents

mTOR1 inhibitor PP242 (4257) was purchased from Tocris; ERK1/2 inhibitor Ulixertinib (BVD-523; HY-15816) was from MedChemExpress. Hoechst 33342 Stain (H3570) was from Thermo Fisher Scientific. PMA (tlrl-pma) was from InvivoGen. Bovine serum albumin (BSA; A4503); sucrose (S7903); imidazole (I2399); human IgG (I4506); human serum (P2918); NOX2 inhibitor: DPI (D2926); NBT (N6876); STING agonist, DMXAA (D5817); zymosan A (Z4250); pHrodo Red zymosan BioParticles Conjugate (P35364); MitoSOX Red mitochondrial superoxide indicator (M36008); HRP (P8375); luminol (A8511); and 4′,6-diamidino-2-phenylindole (DAPI; D9542) were from MilliporeSigma. Latex bead Polybead Microsphere 3.00 µm (17134-15) was from Polysciences. Murine IFN-γ (315-05) was from PeproTech. TRPML1 agonist compound 8 (C8) was provided by Casma Therapeutics. Compounds and drugs were reconstituted as per manufacturer’s recommendation and used as indicated in the text and figure legends.

### Antibodies

Anti-RAB5c [Thermo Fisher Scientific, PA5-39408; RRID: AB_2555998; immunoblotting (IB), 1:2000; immunofluorescence (IF), 1:300], anti–glyceraldehyde-3-phosphate dehydrogenase [clone D16H11; Cell Signaling Technology (CST), 5174T; RRID:AB_10622025; IB, 1:10,000], anti–β-actin (clone 8H10D10; CST, 12262; RRID: AB_2566811; IB, 1:5000), anti–microtubule-associated protein 1 light chain B (CST, 2775; RRID:AB_915950; IB, 1:1000), anti-LC3 (LC3A/LC3B/LC3C; MBL Int., PM036; RRID:AB_2274121; IF, 1:250), anti-RAB5a (CST, 2143; RRID:AB_823625; IB, 1:1000), anti-RAB5b (clone F-9; Santa Cruz Biotechnology, sc373725; RRID:AB_10917429; IB, 1:1000), anti-EEA1 (clone C45B10; CST, 3288; RRID:AB_2096811; IB, 1:1000), anti-UNC93B (Abcam, 69497; RRID:AB_1271408; IB, 1:3000), anti-GM130 (clone 5G8; MBL, M1793; RRID:AB_10694889; IB, 1:500), anti-LAMP1 (clone E5N9Z; CST, 99437; RRID:AB_3065089; IF, 1:150; IB, 1:500), anti-UVRAG (clone D2Q1Z; CST, 13115; RRID:AB_2687988; IB, 1:1000), anti-PI(3)K catalytic subunit type 3 (anti-PIK3C3/VPS34; clone D9A5; CST, 4263; RRID:AB_2299765; IB, 1:1000), anti–Beclin-1 (CST, 3738; RRID:AB_490837; IB, 1:1000), anti-RUBCN (clone D9F7; CST, 8465; RRID:AB_10891617; IB, 1:1000), anti–p-p47phox (S328) (Abcam, 111855; RRID:AB_10860025; IF, 1:50), anti-p47phox (clone erp27205; Abcam, 308256; RRID: not available; IB, 1:1000; IF, 1:250), anti–p-p40phox (Thr^514^) (CST, 4311; RRID:AB_330690; IB, 1:1000), anti-p22phox (Abcam, 75941; RRID:AB_1924907; IB, 1:1000), anti-gp91phox (clone 53; BD Biosciences, 611414; RRID:AB_398936; IB, 1:2000), anti-ERK1/2 (clone 1375F; CST, 4695; RRID:AB_390779; IF, 1:500), anti–p-ERK1/2 (Thr^202/204^) (clone 197G2; CST, 9106; RRID:AB_331768; IF, 1:100), anti-ATP6V0d1 (clone erp18320-38; Abcam, 202899; RRID:AB_2802121; IB, 1:1000), anti-ATP6V1A (clone erp19270; Abcam, 199326; RRID:AB_2802119; IB, 1:4000; IF, 1:250), anti-ATG16L1 (clone 1F12; MBL, M150-3; RRID:AB_1278758; IB, 1:1000), anti-ATG5/12 (clone D5F5U; CST, 12994, RRID:AB_2630393; IB, 1:1000), anti-FLAG (MilliporeSigma, F7425; RRID:AB_439687; IB, 1:2000), anti-GFP (Abcam, 6556; RRID:AB_305564; IB, 1:1000), Alexa Fluor 647 polyclonal goat anti-rabbit IgG (Thermo Fisher Scientific, A21244; RRID:AB_2535812; IF, 1:750), Alexa Fluor 488 polyclonal donkey anti-mouse IgG (Thermo Fisher Scientific, A21202; RRID:AB_141607; IF, 1:750), Alexa Fluor 647 polyclonal donkey anti-mouse IgG (Thermo Fisher Scientific, A31571; RRID:AB_162542; IF, 1:750), HRP-conjugated anti-rabbit IgG (CST, 7074; RRID:AB_2099233; IB, 1:5000), and HRP-conjugated anti-mouse IgG (CST, 7076; RRID:AB_330924; IB, 1:5000) were used as indicated in the manufacturer’s datasheets.

### Mice

C57BL/6J (B6) mice are from the Jackson Laboratory. *Atg16l1*^Δ*WD40*^ mice were previously characterized (*61*). *Rubcn*^−/−^ mice (*4*) were provided by D. Green (St. Jude Children’s Research Hospital, Memphis, TN, USA). *Atg16l1*^Δ*WD40*^ and *Rubcn*^−/−^ mice were previously crossed to C57BL/6 GFP-LC3^Tg^ mice (Noburo Mizushima, University of Tokyo, Tokyo, Japan). Mice were bred and housed in specific pathogen–free facilities at 23°C with a 12-hour light/dark cycle in ventilated cages, with chow and water supply ad libitum at the Animal Research Facilities of the Faculdade de Medicina de Ribeirão Preto, Universidade de São Paulo, Ribeirão Preto, SP. Sex-matched male or female mutant mice and background controls of 8 to 12 weeks of age were used for experiments. The research was conducted in compliance with Ethical Principles in Animal Experimentation adopted by the National Council for Animal Experimentation Control. Experimental protocols were approved by the Ethics Committee on Animal Use of the Faculdade de Medicina de Ribeirão Preto (no. 1208/2023R1).

### Cells

Human embryonic kidney (HEK) 293T (CRL-3216) and Phoenix-Ampho cells (CRL-3113) were obtained from ATCC. RAW264.7 macrophage cell line (ATCC, SC-6003), HEK PEAKrapid cells (ATCC, CRL-2828), and mouse NIH 3T3 fibroblasts expressing human colony-stimulating factor 1 (3T3-MCSF) cells were provided by D. Zamboni (Faculdade de Medicina de Ribeirão Preto). Cells were grown in monolayers in complete high-glucose Dulbecco’s modified Eagle’s medium (DMEM; Gibco, 11995073) supplemented with 10% (v/v) heat-inactivated fetal bovine serum (Gibco, 12657-029), 2 mM l-glutamine (Gibco, 35050061), and penicillin-streptomycin (100 IU/ml; Gibco, 15140122) (cDMEM).

To prepare BMDMs, mice were euthanized, and bone marrow cells were harvested by flushing ethanol-sterilized femurs with DMEM. BMDM differentiation was carried out by culturing cells for 6 to 7 days in cDMEM supplemented with conditioned medium of 3T3-MCSF cells (10%, v/v) and penicillin-streptomycin (100 U/ml). Differentiated BMDMs were harvested by scraping in sterile phosphate-buffered saline (PBS) and seeded onto tissue culture plates in cDMEM 24 hours before stimulation.

All the cell lines were grown at 37°C in a humidified incubator with 5% CO_2_ in air and were periodically confirmed as mycoplasma negative by polymerase chain reaction (PCR). All the reagents used for cell culture were purchased from Thermo Fisher Scientific.

### A. fumigatus

*A. fumigatus* (ATCC46645; WT) or *A. fumigatus* DHN-melanin–deficient derived strain (Δ*pksP*) was grown as previously described (*70*) with slight modifications. Fresh *A. fumigatus* conidia were seeded on yttrium-aluminum-garnet (YAG) medium plates supplemented with malt extract agar [2% (w/v) glucose (MilliporeSigma, G5767), 0.2% (w/v) yeast extract (MilliporeSigma, Y1625), 2% (w/v) malt extract (Gibco, 218630), and 2% (w/v) agar (Oxoid, LP0011B)] and 0.1% (v/v) trace element solution [76.5 mM ZnSO_4_ (221376), 178 mM H_3_BO_3_ (B0394), 40 mM MnCl_2_ (244589), 18 mM FeSO_4_ (F8633), 12.3 mM CoCl_2_ (449776), 10 mM CuSO_4_ (451657), 5.6 mM (NH_4_)_2_MoO_4_ (277908), and 120 mM EDTA (43178); all reagents were from MilliporeSigma] and cultured for 5 days at 37°C. Fungal culture for in vivo experiments was carried out without malt extract. Plates were washed with sterile PBS, followed by centrifugation and filtration through sterile Miracloth (Merck Millipore, 475855) to obtain conidium suspensions. Conidium concentration was determined by light microscopy using a Neubauer chamber.

### Plasmids, cell transfection, and cell transduction

To perform stable heterologous expression of *Rab5* paralogs, a cDNA library generated from mouse placenta total mRNA (Takara, 636672) served as template to amplify the CDS mouse *Rab5a* (NM_025887.4), *Rab5b* (NM_177411.4), and *Rab5c* (NM_024456.5) sequences by PCR, using primers encoding 5′ Mlu I and 3′ Not I restrictions sites. pMX-mCherry-Rab5a, pMX-mCherry-Rab5b, and pMX-mCherry-Rab5c constructs were generated by cloning digested PCR products in-frame with a N-terminal mCherry fusion tag encoded in the MCS of a previously modified pMX-IRES-blasticidin vector (CellBiolabs, no. RTV-016) (*5*). Venus-LC3B and p40^phox^-PX-Venus constructs cloned in modified pMX-IRES-blasticidin vector were previously described (*5*). ATP6V0a3-EGFP constructs cloned in pLXIN were provided by J. P. Luzio (*71*). *Rab5c* (NM_024456.5), its dominant-negative mutant (S35N), and its constitutively active mutant (Q80L), each N-terminally fused to mCherry and cloned into pMMLV-T2A-blasticidin, were custom synthesized by VectorBuilder (Chicago, IL, USA). The pMX-3X-FLAG-Rab5c and pMX-3X-FLAG-mCherry constructs were generated by subcloning *Rab5c* or mCherry sequence digested with 5′ Mlu I and 3′ Not I, into a modified pMX-IRES-blasticidin vector encoding 3X-FLAG sequence inserted in frame upstream of Mlu I. Correct insertion and sequence integrity were confirmed by Sanger sequencing.

To transduce and establish transformed RAW264.7 cell lines, Phoenix-Ampho cells were used to produce vesicular stomatitis virus-G (VSV-G) pseudotyped retrovirus. Phoenix-Ampho cells were transfected with the pMX or pLXIN retroviral vectors harboring the gene constructs of interest using polyethylenimine transfection reagent (PEI-MAX; 1 μg/ml; Polysciences, 23966). Transduction was performed by spinfection (800*g*; 40 min at 37°C) of supernatants from Phoenix-Ampho cells collected at 48 hours posttransfection and filtered through a 0.45-μm membrane (Corning, 431220). Virus-containing cell medium was replenished with fresh complete DMEM supplemented with blasticidin S (pMX; 5 μg/ml; MilliporeSigma, 15205) or geneticin (G418) (pLXIN; Corning, 61-234-RG; 650 mg/ml) 72 hours posttransduction for selection of transformed cells.

To validate the specificity of the commercial antibodies for each *Rab5* paralogs from *Mus musculus*, HEK293T cells were transfect with pMXs-mCherry-Rab5a, pMXs-mCherry-Rab5b, and pMXs-mCherry-Rab5c to overexpress RAB5a, RAB5b, and RAB5c proteins fused with mCherry using PEI-MAX, and cells were harvested 72 hours posttransfection.

For *Rab5* silencing, pLKO.5-puromycin lentiviral constructs encoding shRNA sequences targeting the RAB5 isoforms and a control sequence were purchased from MilliporeSigma: isoform a: sh_*Rab5a* #1: 5′-GCTGGTCAAGAACGGTATCAT-3′ (no. TRCN0000100798) and sh_*Rab5a* #2: 5′-CAAGCAGCCATAGTTGTGTAT-3′ (no. TRCN0000100799); isoform b: sh_*Rab5b* #1: 5′-AGCCAGCCCTAGCATTGTTAT-3′ (no. TRCN0000311442) and sh_*Rab5b* #2: 5′-GGAAGTCTAGCCTGGTGTTAC-3′ (no. TRCN0000381840); isoform c: sh_*Rab5c* #1: 5′-GCTAAGAAGCTTCCCAAGAAT-3′ (no. TRCN0000100749) and sh_*Rab5c* #2: 5′-GCAATGAACGTGAATGAAATT-3′ (no. TRCN0000100747); and control shRNA (sh_Ctrl): 5′-CCGGCAACAAGATGAAGAGCACCAACTCGAGTTGGTGCTCTTCATCTTGTTGTTTTT-3′ (no. SHC202).

To produce lentiviral particles, HEK PEAKrapid cells were transfected with pVSV-G (pMD2.G; Addgene, no. 12259), pPAX2 (Addgene, no. 12260), and pLKO.5 using PEI-MAX. For RAW264.7 cells, transduction with filtered lentiviral particles was carried out as described above. Cell medium was replenished at 72 hours posttransduction with fresh medium containing puromycin (5 μg/ml; MilliporeSigma, P8833) to select transformed cells. For BMDMs transduction, nonadherent bone marrow cells were collected at day 2 of differentiation and mixed with the HEK PEAKrapid supernatants containing the lentiviral particles. Medium was replenished 72 hours posttransduction (day 5 of differentiation) with fresh warm medium containing puromycin (5 μg/ml); selected BMDMs were collected at day 7 and seeded for further experiments.

For the deletion of *Rab5c* in RAW264.7 cells by CRISPR-Cas9, guide sequences targeting the second exon of *Rab5c* from *M. musculus* (NM_024456.5) were designed using CRISPick design tool (https://portals.broadinstitute.org/gppx/crispick/public). Two sequences were selected [guide RNA (gRNA) #1: 5′-CCACGCTCACTGGTACTACT-3′ and gRNA #2: 5′-CCAGGAGAGCACAATTGGAG-3′] and cloned into the lentiCRISPR-v2GFP backbone (gift from B. Stringer, Griffith University, Queensland, Australia; Addgene, no. 82416) digested with Bsm BI (Thermo Fisher Scientific, ER0451). To produce lentiviral particles, HEK PEAKrapid cells were transfected using PEI-MAX with pMD2.G, pPAX2, and LentiCRISPR-v2GFP constructs. Transduction of lentiviral particles was carried out as described above. After 48 hours of lentiviral transduction, a pool of GFP cells was selected by cell sorting (BD FACSAria Fusion Flow Cytometer, BD Biosciences) and expanded for further experiments.

### Fluorescent microscopy

For fluorescent live-cell imaging, RAW264.7 cells (0.5 × 10^5^ to 1.5 × 10^5^ per plate) were seeded onto 35-mm glass-bottomed dish (MatTek, P35G-1.5-14-C) in 2 ml of cDMEM and cultivated overnight in the presence of IFN-γ (200 U/ml) before imaging. Op-zym particles were generated by incubating zymosan A from *Saccharomyces* (1 × 10^6^ zymosan particle/ml) with human serum (v/v) for 30 min at 37°C. The solution was then centrifuged at 4000*g* for 5 min and resuspended in sterile PBS at 10 mg/ml. The solution was syringe homogenized several times using a 26-gauge needle to break up aggregates. Plates were mounted on an Olympus SpinSR confocal microscope equipped with an Olympus IX83 stand, Olympus 60× 1.5–numerical aperture UPLAPO objective lens, Yokogawa CSU-W1 scan head, and a Hamamatsu Orca Fusion camera. Images were acquired with a 2 × 2 camera bin and a 108-nm pixel size. Laser excitation and emission filters for the mCherry and Venus channels were 561 nm [excitation (ex)] and 617/73 nm [emission (em)] and 488 nm (ex) and 525/50 nm (em), respectively. The image acquisition software was Olympus cellSens v4.1. All imaging with live cells was performed within incubation chambers at 37°C and 5% CO_2_. For mCherry-RAB5c, mCherry-LC3B, p40^phox^-PX-Venus, and ATP6V0a3-EGFP imaging, *z* stacks were acquired every 30 s following the addition of Op-zym particles at a 1:4 macrophage:particle ratio. ImageJ software (*72*) was used to measure the mean intensity of fluorescence in a region of interest (ROI) surrounding an individual Op-zym particle and divided by the intensity of a ROI in the cytosol. The real-time images of RAW264.7 cells expressing ATP6V0a3-EGFP were also deconvolved using Huygens Professional Deconvolution Software (v21.04, Scientific Volume Imaging, Hilversum, The Netherlands).

For fixed immunofluorescence confocal microscopy, 2.5 × 10^5^ RAW264.7 cells or BMDMs were seeded onto untreated 13-mm round coverslip in cDMEM (placed at the bottom of the well of a 24-well plate) and preincubated with IFN-γ (200 U/ml) for 16 hours. Cells were fed Op-Zym or *A. fumigatus* for 25 min (unless stated otherwise in the legend), then rinsed twice with PBS, and fixed for 20 min with 4% (w/v) paraformaldehyde (PFA; MilliporeSigma, P6148) in PBS. Cells were quenched with 0.1 M glycine (MilliporeSigma, G7126) in PBS for 5 min. Samples that required immunostaining were permeabilized with 0.03% Triton X-100 (MilliporeSigma) for 10 min or with warm digitonin (200 mg/ml) in PBS for 13 min. For immunolabeling of p-p47^phox^, p47^phox^, and ATP6V1A, cells were fixed and permeabilized with 100% ice-cold methanol (Sigma-Aldrich, 179337) for 5 min at −20°C. Next, cells were rinsed twice in PBS and incubated for 1 hour at room temperature (RT) in PBS containing 1% (w/v) BSA and normal donkey IgG (5 μg/ml; Jackson ImmunoResearch Laboratories Inc., 017-000-003). Cells were labeled with primary antibodies diluted in PBS containing 1% (w/v) BSA for 1 hour at RT or overnight at 4°C. Next, cells were rinsed thoroughly in PBS and incubated for 30 min at RT with the secondary antibodies diluted in PBS. For nuclear staining, after incubation with secondary antibodies, the cells were incubated for 5 min at RT with DAPI (0.2 μg/ml) or Hoechst 33342 (2 μM) in PBS. Cells were then rinsed in PBS and then in ddH_2_O and mounted onto microscope slides with Fluoromount-G (Invitrogen, 00-4958-02) or with ProLong Gold Antifade Mountant (Invitrogen, P36934). Cells incubated without primary antibody served as negative controls. Samples were analyzed using a Zeiss LSM 780 laser scanning confocal microscope (Carl Zeiss Ltd.) using Zen software (Carl Zeiss Ltd.) or a LEICA STELLARIS 8 laser scanning confocal microscope acquired or not with LIGHTNING enhanced resolution (Leica Microsystems) using LAS X Life Science software and plugin three-dimensional viewer (modes of volume, blend, and surface) (Leica Microsystems). High-resolution images were acquired using a Nikon Eclipse Ti2-E A1 high-resolution microscope (Nikon Instruments Inc.) using Nikon Elements software (Nikon Instruments Inc.). The mean fluorescence intensity (MFI) of the target protein on individual phagosomes was determined using ImageJ software. A mask drawn around the edge of a sampled phagosome and an adjacent of 0.6 or 0.5-μm band were considered ROI to calculate the fluorescence intensity. For each condition, at least five images and 40 phagosomes were quantified per experiment. For BMDMs stimulated with *A. fumigatus*, phagosomes surrounded by a fluorescence rim of the indicated target protein were scored as positive. At least 50 phagosomes were analyzed for each condition per experiment, in a blinded fashion by the same investigator.

### Correlative light–electron microscopy

CLEM experiments were conducted using an adapted protocol designed for the preparation of samples for focused ion beam–scanning electron microscopy (FIB-SEM) experiments by using the Megametal (rOTO + UA + Pb) resin embedding technique. First, 0.5 × 10^5^ to 1.5 × 10^5^ RAW264.7 cells expressing mCherry-RAB5c seeded on 35-mm glass-bottomed dish (MatTek, P35G-1.5-14-C-GRD.s), pretreated with IFN-γ in 2 ml of cDMEM and fed Op-zym as described above, were live imaged up to the point of clear RAB5c recruitment. Cells were then promptly fixed by addition of methanol free formaldehyde (TAAB Laboratory and Microscopy, F017/3) to the medium for final concentration of 2% (v/v) and incubated for 1 hour at RT. Next, samples were thoroughly washed in 0.1 M phosphate buffer (MilliporeSigma, P5244), followed by en bloc staining according to the Ellisman protocol (www.protocols.io/view/preparation-of-biological-tissues-for-serial-block-36wgq7je5vk5/v2). Briefly, samples were incubated in 2 M phosphate buffer containing 2% (v/v) osmium tetroxide (Agar Scientific, R1023) and 1.5% (v/v) potassium ferrocyanide (MilliporeSigma, P8131) for 1 hour at 4°C, washed in ddH_2_O, and incubated in 1% thiocarbohydrazide (MilliporeSigma, 223220) for 20 min. Then, samples were washed with ddH_2_O, incubated in 2% osmium tetroxide for 30 min, washed once again with ddH_2_O, and incubated overnight in 2% uranyl acetate (TAAB Laboratory and Microscopy, U007) at 4°C. The next day, samples were stained en bloc with Walton’s lead aspartate solution [AgNO_3_ (S6506), l-aspartic acid (A7219), and NaOH (S5881); MilliporeSigma] for 30 min at 60°C, washed with ddH_2_O, and dehydrated in graded ethanol (VWR Ethanol Absolute ≥99.8%, AnalaR NORMAPUR; VWR, 20821.330) followed by propylene oxide (MilliporeSigma, 110205) for 10 min at RT. Samples were embedded in Hard-Plus Resin 812 (Science Services, E14115), mounted on SEM stubs using silver epoxy resin (Agar Scientific, AGG3349), and coated with a 5-nm layer of platinum in a CU10 coater (Safematic, Switzerland).

For FIB-SEM, the specimens were placed into a Zeiss Crossbeam 550 scanning electron microscope running Atlas Engine (Zeiss, Oberkoch, Germany). The previously identified ROI was relocated in the SEM by imaging at 2 and 10 kV. Once positioned, the ROI was prepared for imaging by depositing tracking marks and milling a trench with the FIB, thereby exposing a face suitable for SEM imaging. Electron micrographs were obtained with isotropic 10-nm resolution with a 9- or 10-μs dwell time. The SEM was operated at 2-kV accelerating voltage and 500-pA current. To improve signal to noise ratio, both backscatter and secondary electron data were collected. Ion beam milling was performed at an accelerating voltage of 30 kV and current of 700 pA. For image analysis, image stacks were initially aligned using Atlas 5 software, before the combined image channels were inverted and exported as a single stack. Aligned image stacks were further aligned using drift correction and Alignment to Median Smoothed Template with a median size of 15 in Microscopy Image Browser (*73*) and were cropped to remove edge effects. Electron and fluorescent image overlays were generated using the CLEM plugin in Icy software.

### Immunoblotting

Cells (1 × 10^6^ to 3 × 10^6^) were washed twice with ice-cold PBS and immediately lysed with ice-cold radioimmunoprecipitation assay (RIPA) buffer [50 mM tris-HCl (pH 7.4; T6066), 150 mM NaCl (31434), 1 mM EDTA (E9884), 1% (v/v) Triton X-100 (T8787), 0.1% (w/v) SDS (L3771), and 0.5% (w/v) sodium deoxycholate (D6750); all reagents were from MilliporeSigma] supplemented with protease (cOmplete; Roche, 11836153001) and phosphatase inhibitors (PhosSTOP; Roche, 04906837001). Lysates were centrifuged at 16,000*g* at 4°C for 20 min, and the supernatants were collected. The protein content was quantified using the Pierce Bradford Protein Assay Kit (MilliporeSigma, 23200), with BSA as the standard. Lysate containing 30 μg of protein was mixed with NuPAGE LDS Sample Buffer (Thermo Fisher Scientific) supplemented with 10% (v/v) 2-β-mercaptoethanol (MilliporeSigma, M3138) and heated for 8 min at 90°C, and the proteins were separated electrophoretically by SDS–polyacrylamide gel electrophoresis (PAGE) using 4 to 12% Criterion XT Bis-Tris precast gels (Bio-Rad, 34501233/4/5) and 10 and 18% (v/v) polyacrylamide SDS-PAGE gels (MilliporeSigma, A3574) and transferred to 0.45-μm polyvinydine difluoride membranes (Merck Millipore, IPVH00010). Membranes were blocked for 1 hour at RT in TBS-T [50 mM tris-HCl (pH 7.5), 150 mM NaCl, and 0.05% (v/v) Tween 20 (P9416)] supplemented with 4% (w/v) BSA and incubated overnight at 4°C or 1 hour at RT with the individual primary antibodies diluted in blocking buffer. The membranes were then washed, incubated for 30 min at RT with the appropriate anti-IgG conjugated to HRP in TBS-T, washed and developed using SuperSignal West Pico Plus Chemiluminescent Substrate (Thermo Fisher Scientific) in an MI-5 X-Ray film processor (Medical Index) or with ChemiDoc XRS+ (Bio-Rad Laboratories Inc.). After exposure, the films were digitized, and the mean optical density of the target protein was determined using ImageJ software. For stripping, after the first immunoblotting, the membrane was immersed in Restore Western blot Stripping Buffer (Thermo Fisher Scientific, 21059) for 15 min at RT and washed, and the immunoblotting was repeated as described above.

### Phagosome purification

RAW264.7 cells were seeded onto 150-mm tissue-treated plate (Corning, 430599; 2 × 10^7^ cells per plate) in cDMEM and incubated for 16 hours in the presence of IFN-γ (200 U/ml). Cells were fed IgG-beads (human IgG–coated latex beads) or BSA-beads (BSA-coated latex beads) at 1:8 macrophage:bead ratio for 40 min and purified as previously described (*5*). Briefly, plates were extensively rinsed with ice-cold PBS to eliminate noninternalized beads. Cells were scraped in 10 ml of ice-cold PBS, pelleted, and mechanically homogenized in 1 ml of 8.5% (w/v) sucrose (250 mM) through a 27-gauge needle. The cell homogenate was mixed with 62% (w/v) sucrose (181 mM) to reach a final sucrose concentration of ~42%; layered onto 62% sucrose; and carefully topped with 35% (w/v; 102 mM), 25% (w/v; 73 mM), and 10% (w/v; 29 mM) sucrose solutions in sequence in polycarbonate centrifuge tubes. Sucrose-containing buffers were prepared in 3 mM imidazole (pH 7.4). Following ultracentrifugation at 100,000*g* for 1 hour at 4°C, phagosome-containing beads were collected at the 10 to 25% sucrose interface. Purified bead-containing phagosomes were washed with ice-cold PBS, pelleted by ultracentrifugation at 100,000*g* for 20 min at 4°C, and resuspended in lysis buffer [50 mM tris-HCl (pH 7.5), 150 mM NaCl, 5 mM EDTA, and 1% (v/v) Igepal-CA-630 (13021); all reagents were from MilliporeSigma] supplemented with protease inhibitor (cOmplete). Equal volume per sample was analyzed by SDS-PAGE and immunoblotting.

### Analysis of phagolysosome maturation

For phagosome acidification assays, RAW264.7 cells were seeded onto 24-well plates (3.0 × 10^5^ cells per well) in cDMEM for 16 hours in the presence of IFN-γ (200 U/ml). Cells were then fed the pH-sensitive probe pHrodo Red Op-zym particles (1:2 macrophage:particle ratio) for 45 min. Cells were rinsed three times with ice-cold PBS, scraped, centrifuged at 300*g* for 5 min, and resuspended in fluorescence-activated cell sorting (FACS) buffer [1% (w/v) BSA and 1 mM EDTA 1 mM in PBS]. Sample acquisitions were performed in a FACSVerse (BD Biosciences) flow cytometer, and data were analyzed with FlowJo v10.8 Software. Time-lapse microscopy assessment of acidification was performed in RAW264.7 cells (2.5 × 10^5^ cells per well) seeded onto dish (MatTek, 80411) in cDMEM and incubated for 16 hours. The media were then replenished with fresh DMEM, containing high glucose and Hepes and without phenol red (Gibco, 21063029). Chambers were transferred to a Nikon BioStation IM-Q equipped with CELL-S2, cells were fed the same particles, and two images for each condition were recorded every 340 s. Data were analyzed with ImageJ software.

For the analysis of proteolytic degradation, RAW264.7 cells (1.5 × 10^5^ per plate) were seeded onto 35 mm glass-bottomed dishes (MatTek, P35G-1.5-14-C) in 2 ml of cDMEM and cultured overnight in the presence of IFN-γ (200 U/ml). To prepare IgG-opsonized DQ-BSA–coated beads, latex beads were incubated with DQ Green BSA (Invitrogen, D12050) at RT with shaking at 950 rpm for 2 hours. Beads were subsequently washed three times with PBS and then opsonized with human IgG at 37°C and 950 rpm rotation for 30 min. Beads were then washed three times with PBS and syringe homogenized several times using a 26-gauge needle to break up aggregates before addition to the cells. Cells were pretreated or not for 45 min with pepstatin A (10 μg/ml; MilliporeSigma, EI10) and leptin (MilliporeSigma, L5037), washed with cold PBS, and fed IgG-opsonized DQ-BSA–coated beads in cDMEM for 5 min at 4°C. Then, cells were washed twice with warm PBS, the cDMEM was replenished, and the samples were incubated at 37°C for 1 hour before imaging. The data were acquired using LEICA STELLARIS 8 laser scanning confocal microscope (Leica Microsystems). The MFI of internalized DQ-BSA-beads within individual phagosomes was determined using ImageJ software, and the DQ-BSA signal from noninternalized beads was subtracted as baseline background. In each independent experiment, >97 phagosomes per genotype were analyzed.

### ROS detection

For the NBT assay, RAW264.7 cells or BMDMs were seeded onto untreated 13-mm round coverslip in cDMEM (placed at the bottom of the well of a 24-well plate) and cultivated in the presence of IFN-γ (200 U/ml) for 16 hours. Before stimulation, cells were incubated with NBT in RPMI medium (Gibco, 11835030) at 0.04 mg/ml for 10 min at 37°C. Where indicated, cells were also preincubated with ERK1/2 inhibitor before stimulation for 25 min. RAW264.7 cells were stimulated with Op-zym (1:8 macrophage:particle ratio) for 25 min or with opsonized sheep red blood cell (sRBC; 1:8 macrophage:particle ratio) prepared as previously described (*74*, *75*). In brief, 100 μl of sRBC (10% suspension; Stratech, ISHRBC10P-MIN) was sedimented at 500*g* for 1 min and washed twice with PBS. Resuspended pellet was incubated with 10 μl of rabbit IgG fraction to sRBC (Fisher Scientific, ICN55806) and rotated gently for 50 min at 37°C. The pellet was washed three times with PBS and spun down at 500*g* for 1 min. The pellet was resuspended in a final volume of 1 ml of PBS and used for the further experiments. BMDMs were stimulated with *A. fumigatus* melanin-deficient Δ*pksP* strain (1:4 macrophage:particle ratio) for 45 min. Samples were then fixed with 100% ice-cold methanol for 5 min at −20°C, extensively rinsed with PBS, once with distilled H_2_O, and mounted onto microscope slides with Fluoromount-G. Slides were recorded by differential interference contrast or bright-field imaging on a Nikon Eclipse Ti2-E microscope (Nikon Instruments Inc.).

The luminol assay was performed as previously described (*21*). Briefly, RAW264.7 cells (1 × 10^5^ cells per well) were seeded in cDMEM in 96-well white microwell plates (Thermo Fisher Scientific, 136101) and incubated in the presence of IFN-γ (200 U/ml) for 16 hours. Cells were then rinsed with PBS, and cell medium was replenished with sterile DPBS (Dulbecco’s PBS with Ca^2+^ and with Mg^2+^; MilliporeSigma, D8662) containing HRP (0.32 U/ml), luminol (125 nM), 0.1% (w/v) glucose (MilliporeSigma, G8769), and sodium bicarbonate (4 mM; MilliporeSigma, S8761). Cells were incubated for 3 min at 37°C before stimulation. In some experiments, DPI was added to cell medium 7 min before the incubation with the luminol/HRP solution and maintained until the end of the assay. Stimuli [Op-zym, IgG-beads, and BSA-beads (1:8 macrophage:particle ratio) or PMA (500 ng/ml)] were added to the wells and measurements were obtained immediately in the MicroLumatPlus LB 96 V (Berthold technologies) at 37°C.

To analyze mitochondrial ROS generation, RAW264.7 cells (3.0 × 10^5^ cells per well) were seeded in 24-well plates and incubated for 16 hours in the presence of IFN-γ (200 U/ml). Cells were then washed twice with warm PBS and incubated with a prewarmed MitoSOX Red mitochondrial superoxide indicator at 2.5 μM in serum-free DMEM for 30 min before stimulation. Cells were fed Op-zym (1:8 macrophage:particle ratio) for 30 min, rinsed with warm PBS, scraped from plates, centrifuged at 300*g* for 5 min, and resuspended in FACS buffer. Cells were analyzed by flow cytometry (BD Biosciences, BD LSRFortessa Cell Analyzer), and data analysis was performed using FlowJo v10.8 Software.

### Anti-FLAG immunoprecipitation (EndoIP and co-IP)

RAW2647 cells (5.0 × 10^6^) coexpressing ATP6V0a3-EGFP and either 3X-FLAG-RAB5c or 3X-FLAG-mCherry were seeded in three 10-cm plates (EndoIP) or two 10-cm plates (co-IP) per replicate and incubated for 16 hours in the presence of IFN-γ (200 U/ml). The immunoprecipitations were performed as previously described (*58*). Briefly, cells were placed on ice, washed with ice-cold PBS, harvested in DPBS, and pelleted at 500*g* for 4 min at 4°C. The supernatant was removed, and cells were resuspended in 1.5 ml of KPBS [100 mM KH_2_PO_4_ (P3786) and 25 mM KCl (P5405) (pH 7.25), all reagents were from MilliporeSigma] supplemented with protease and phosphatase inhibitors (iKPBS). Cells were then lysed on ice with 50 strokes using a 2-ml Douce homogenizer and a Potter-Elvehjem PTFE pestle and glass tube (MilliporeSigma, P7984). Lysates were centrifuged at 1000*g* for 4 min at 4°C. The postnuclear supernatants (PNS; 100 μl) were retained as input lysate. The remaining PNSs were transferred to 175 μl of prewashed anti-FLAG M2 magnetic beads (MilliporeSigma, M8823) and incubated at 4°C with gentle rotation for 50 min. After incubation, flow-through was collected, combined with RIPA buffer, and reserved for immunoblotting. Anti-FLAG beads were washed three times with 1 ml of iKPBS; and bounded proteins were eluted with 120 μl of 0.5% (v/v) Igepal-CA-630 in iKPBS, combined with 40 μl of 4× NuPAGE LDS Sample Buffer supplemented with 10% (v/v) 2-β-mercaptoethanol, heated at 90°C for 8 min, and processed for immunoblotting.

For anti-FLAG immunoprecipitation, cells were washed with ice-cold PBS, scraped, pelleted, and lysed on ice with 1 ml of Pierce IP Lysis Buffer (Thermo Fisher Scientific, 87788) supplemented with protease and phosphatase inhibitors. Samples were centrifuged at 20,000*g* for 15 min, and the supernatant was incubated with 150 μl of prewashed anti-FLAG M2 magnetic beads at 4°C with gentle rotation for 50 min. Subsequent steps were performed as described above.

### *A. fumigatus*–killing assay

BMDMs seeded in 96-well plates (1 × 10^5^ cells per well) were stimulated with *A. fumigatus* conidia at a 10:1 macrophage:conidium ratio. After 1 hour of incubation, cells were rinsed three times with warm sterile PBS to remove nonadherent, nonphagocytosed conidia, and medium was replenished with fresh cDMEM. Where indicated, DPI was added 1 hour before infection (5 μM final concentration). At 1 or 6 hours postinfection, cells were lysed with sterile ddH_2_O, and cell lysate were seeded onto YAG-agar plates. Colony-forming units (CFU) were counted after 24 hours of incubation at 37°C. Conidium viability was determined as the ratio CFU at 6 hours/CFU at 1 hour.

### In vivo infection with *A. fumigatus*

Mice were identified by ear tag, but experimenters were not blinded to their genotype. No statistical tests were used to determine sample size. Treatments were randomized within cages. Mice were anesthetized with intraperitoneal injection of a solution of ketamine chloride (180 mg/kg) and xylazine chloride (20 mg/kg) and each challenged by intranasal instillation with vehicle (PBS) or 1 × 10^9^ *A. fumigatus* conidia in 20 μl of PBS. At 72 hours postinfection, mice were euthanized, and the bronchoalveolar fluid (BAL) was collected in 1 ml of lavage with sterile PBS. The BAL (100 μl) was plated on YAG agar for CFU counts. The remaining fluid was cleared by centrifugation, and the supernatants were used for albumin quantification by a colorimetric assay (Labtest, no. 19-1/250) or IL-6 (DY406), CCL2 (DY479), TNF-α (DY410), and IL-1β (DY401) dosing by enzyme-linked immunosorbent assay (ELISA). All ELISA kits were acquired from R&D Systems and used according to the procedures supplied by the manufacturer. The lungs were harvested, then fixed in PBS with 4% PFA for 4 hours, and subsequently transferred to 70% ethanol for at least 24 hours before paraffin embedding. Serial sections (5 μm) were stained with hematoxylin and eosin or Grocott-Gomori’s methenamine silver and coverslips were mounted with Permount. Images were captured using an Olympus BX61 Motorized Slide Scanner Microscope Pred VS120 (Olympus, Hamburg, Germany).

### Data quantification and statistical analysis

Data were plotted and analyzed with GraphPad Prism version 10.3.0 (GraphPad Software, Boston, MA, USA; www.graphpad.com). Please refer to the legend of the figures for description of samples (mice, cells, or experimental replicates) and sample sizes. No statistical tests were used to estimate sample size. Statistical significance was calculated with either unpaired two-tailed Student’s *t* test or analysis of variance (ANOVA), as specified in the legend of the figures. All figures were generated using BioRender.com (available online at https://biorender.com).
