# Supplementary material for: RAB5c orchestrates LC3-associated phagocytosis to promote microbicidal function of macrophages
Source: Sci Adv. 2026 May 8;12(19):eadz0196. doi: 10.1126/sciadv.adz0196 (PMC13155314; doi:10.1126/sciadv.adz0196)
Supplement: Supplementary file 1 — Figs. S1 to S4 Legends for movies S1 to S4 [file sciadv.adz0196_sm.pdf]

Science Advances
AAAS

# Supplementary Materials for

## RAB5c orchestrates LC3-associated phagocytosis to promote microbicidal function of macrophages

Edismauro Garcia Freitas-Filho *et al.*

Corresponding author: Oliver Florey, oliver.florey@babraham.ac.uk; Larissa Dias Cunha, larissacunha@usp.br

*Sci. Adv.* **12**, eadz0196 (2026)
DOI: 10.1126/sciadv.adz0196

**The PDF file includes:**

Figs. S1 to S4
Legends for movies S1 to S4

**Other Supplementary Material for this manuscript includes the following:**

Movies S1 to S4

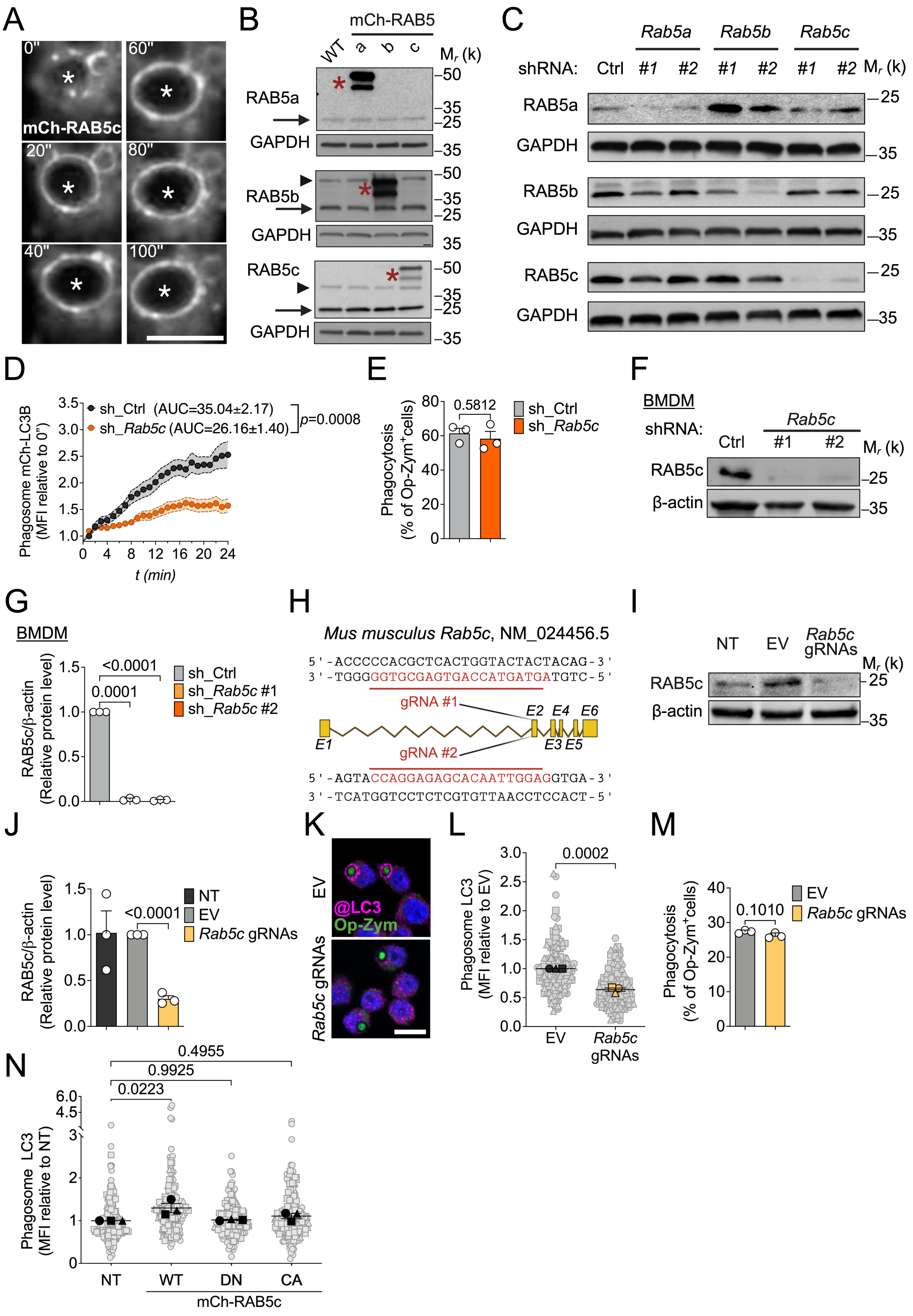

A
0''
60''
mCh-RAB5c
20''
80''
40''
100''
B
mCh-RAB5
WT
a
b
c
Mr (k)
RAB5a
GAPDH
RAB5b
GAPDH
RAB5c
GAPDH
C
Rab5a
Rab5b
Rab5c
shRNA:
Ctrl
#1
#2
RAB5a
GAPDH
RAB5b
GAPDH
RAB5c
GAPDH
D
sh_Ctrl (AUC=35.04±2.17)
sh_Rab5c (AUC=26.16±1.40)
p=0.0008
Phagosome mCh-LC3B
(MFI relative to 0'')
t (min)
E
0.5812
sh_Ctrl
sh_Rab5c
Phagocytosis
(% of Op-Zym+cells)
F
BMDM
shRNA:
Ctrl
Rab5c
#1
#2
RAB5c
β-actin
G
BMDM
<0.0001
0.0001
sh_Ctrl
sh_Rab5c #1
sh_Rab5c #2
RAB5c/β-actin
(Relative protein level)
H
Mus musculus Rab5c, NM_024456.5
5'-ACCCCCACGCTCACTGGTACTACTACAG-3'
3'-TGGGGGTGCGAGTGACCATGATGATGTC-5'
gRNA #1
E1
E2
E3
E4
E5
E6
gRNA #2
5'-AGTACCAGGAGAGCACAATTGGAGGTGA-3'
3'-TCATGGTCCTCTCGTGTTAACCTCCACT-5'
I
NT
EV
Rab5c gRNAs
Mr (k)
RAB5c
β-actin
J
<0.0001
NT
EV
Rab5c gRNAs
RAB5c/β-actin
(Relative protein level)
K
EV
@LC3
Op-Zym
Rab5c gRNAs
L
0.0002
Phagosome LC3
(MFI relative to EV)
EV
Rab5c gRNAs
M
0.1010
EV
Rab5c gRNAs
Phagocytosis
(% of Op-Zym+cells)
N
0.4955
0.9925
0.0223
Phagosome LC3
(MFI relative to NT)
NT
WT
DN
CA
mCh-RAB5c

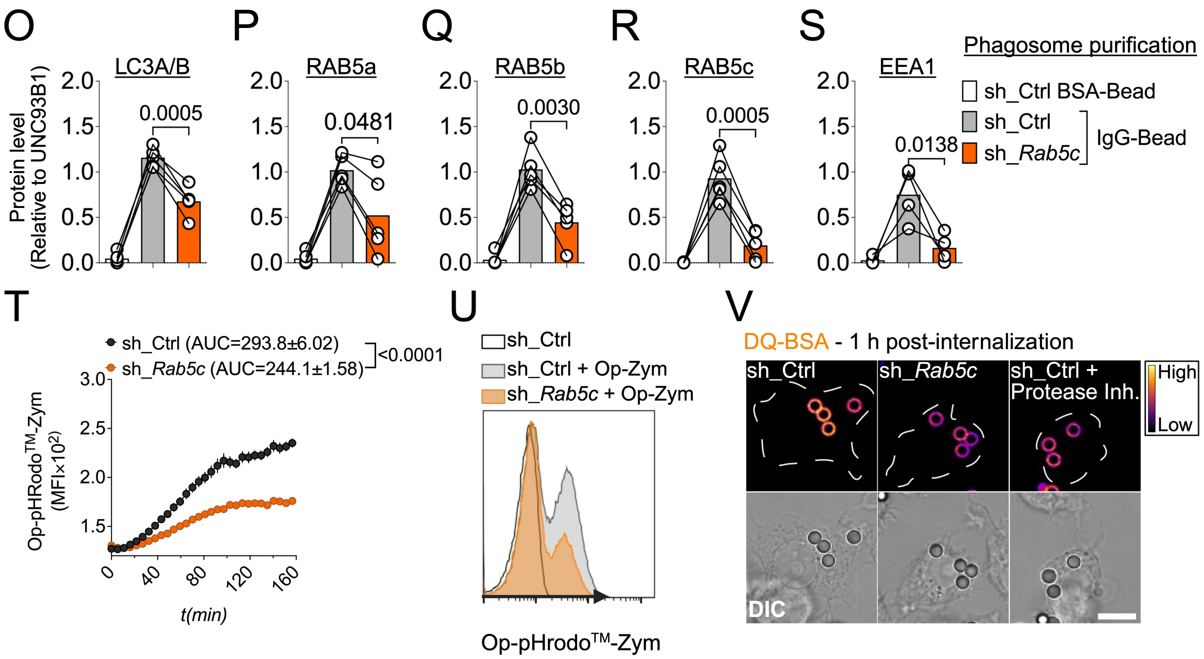


**Fig. S1.**

**Related to Figure 1.** (**A**) Time-lapse confocal images of mCh-RAB5c at a phagosome (*) of RAW264.7-cells fed Op-zym. Scale bar: 5 μm (*related to Movie S1*). (**B**) Immunoblot of HEK293T cells [non-transduced (NT) or expressing the indicated constructs] probed with isoform-specific anti-RAB5 antibodies; GAPDH, loading control. *: heterologous expressed RAB5. Arrows: endogenous isoforms. Arrowheads: nonspecific bands. (**C**) Immunoblot of RAW264.7 cells expressing sh_Ctrl or indicated shRNA sequences. GAPDH, loading control. (**D**) mCh-LC3B MFI at Op-zym$^{+}$ phagosomes (n=11) of RAW264.7 cells expressing sh_Ctrl or sh_*Rab5c* (*related to Movie S2*). (**E**) Percentage of Op-zym phagocytosis, assessed by immunofluorescence of RAW264.7 cells expressing sh_Ctrl or sh_*Rab5c*. (**F** and **G**) Immunoblot (F) and quantification (G) of RAB5c in BMDM expressing sh_Ctrl or sh_*Rab5c*. β-actin, loading control. (**H**) *Rab5c locus* (*Mus musculus*) showing CRISPR target sites. Created in BioRender. Cunha, L. (2026) https://BioRender.com/5skzhel. (**I** and **J**) Immunoblot (I) and quantification (J) of RAB5c in non-transduced (NT) RAW264.7 cells, or cells transduced with empty vector (EV) or *Rab5c* gRNAs. β-actin, loading control. (**K** to **M**) Confocal images (K), LC3 MFI at phagosomes (L), and percentage of phagocytosis (M) in RAW264.7 cells expressing EV or *Rab5c* gRNAs fed Op-zym. Scale bar: 10 μm. (**N**) LC3 MFI at phagosomes in (NT) RAW264.7 cells, or cells expressing mCh-RAB5c (WT); dominant-negative mCh-RAB5c(S35N), or constitutively active mCh-RAB5c(Q80L), and fed Op-zym. (**O** to **S**) Quantification of LC3A/B (O), RAB5a (P), RAB5b (Q), RAB5c (R), and EEA1 (S) in purified phagosomes; UNC93B, loading control (*related to Fig. 1I*). (**T**) Op-pHrodo™-zym MFI (time-lapse epifluorescence microscopy) in RAW264.7 cells expressing sh_Ctrl or sh_*Rab5c*. (**U**) Histogram of Op-pHrodo™-zym uptake in RAW264.7 cells, assessed by flow cytometry (*related to Fig. 1 L and M*). (**V**) Confocal images (shown as FIRE LUT) of RAW264.7 cells ± protease inhibitors fed opsonized-DQ-BSA-coated beads. Scale bar: 10 μm (*related to Fig. 1N*). Colored symbols (D, L, N, T) or bars (E, G, J, M, O to S) represent means of biological replicates, each indicated as a gray (phagosome) or white (sample) object. Shaded area (D) or error bars, ± S.E.M. Statistical comparison are unpaired Student's *t*-test or ANOVA and Tukey's multiple

comparisons (N). Data pooled from 3 (J, L, M, N, T) or 4-5 (O to S) independent experiments, or representative of 2 (A to F, J, U), or 3 (G, I, K, T) independent experiments.

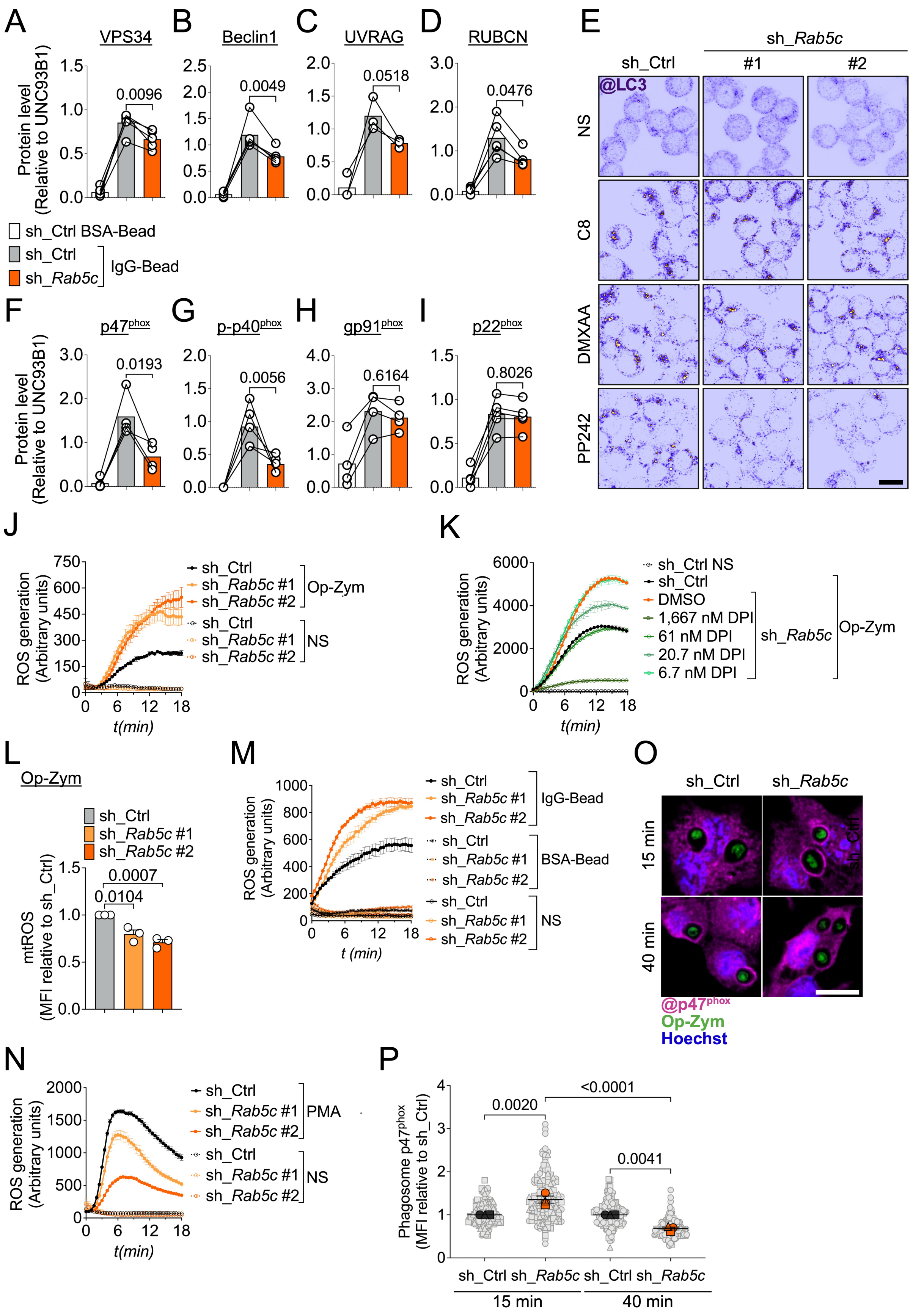
A
VPS34
Protein level (Relative to UNC93B1)
0.0096
B
Beclin1
0.0049
C
UVRAG
0.0518
D
RUBCN
0.0476
sh_Ctrl BSA-Bead
sh_Ctrl
sh_Rab5c
IgG-Bead
E
sh_Ctrl
sh_Rab5c
#1
#2
@LC3
NS
C8
DMXAA
PP242
F
p47phox
0.0193
G
p-p40phox
0.0056
H
gp91phox
0.6164
I
p22phox
0.8026
J
ROS generation (Arbitrary units)
sh_Ctrl
sh_Rab5c #1
sh_Rab5c #2
Op-Zym
NS
t(min)
K
sh_Ctrl NS
sh_Ctrl
DMSO
1,667 nM DPI
61 nM DPI
20.7 nM DPI
6.7 nM DPI
sh_Rab5c
Op-Zym
L
Op-Zym
mtROS (MFI relative to sh_Ctrl)
0.0007
0.0104
M
IgG-Bead
BSA-Bead
t (min)
O
15 min
40 min
@p47phox
Op-Zym
Hoechst
N
PMA
P
Phagosome p47phox (MFI relative to sh_Ctrl)
0.0020
<0.0001
0.0041
15 min
40 min

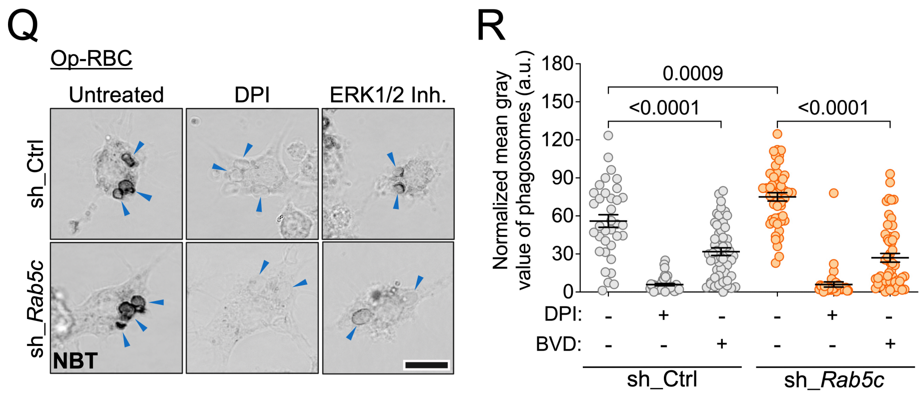


**Fig. S2.**

**Related to Figure 2.** RAW264.7 cells or BMDM expressing sh_Ctrl or sh_*Rab5c*. (**A** to **D**) Quantification of VPS34 (A), Beclin1 (B), UVRAG (C), and RUBCN (D) in purified phagosomes. UNC93B1, loading control (*related to Fig. 2A*). (**E**) Confocal images (shown as ICA LUT) of LC3 in RAW264.7 cells non-stimulated (NS) or treated as in *Fig. 3D*. (**F** to **I**) Quantification of $p47^{phox}$ (F), p-$p40^{phox}$ (G), $gp91^{phox}$ (H), and $p22^{phox}$ (I) in purified phagosomes. UNC93B1, loading control (*related to Fig. 2F*). (**J** and **K**) Chemiluminescence assessment of luminol oxidation in RAW264.7 cells non-stimulated or fed Op-zym (n=3 samples). In (K), cells were pre-treated ± DPI at the indicated concentrations. (**L**) MitoSOX Red MFI (mtROS) in RAW264.7 cells fed Op-zym. **(M** to **N**) Chemiluminescence assessment of luminol oxidation in RAW264.7 cells stimulated with IgG- or BSA-beads (M), and PMA (500 µg/mL) (N) (n=3 samples). (**O** and **P**) Confocal images (O) and MIF (P) of $p47^{phox}$ at phagosome in RAW264.7 cells fed Op-zym for 15 or 40 min. (**Q** and **R**) Bright field images (Q) and quantification (R) of NBT deposits in opsonized-red blood cells $(RBC)^{+}$ phagosomes in RAW264.7 cells ± DPI (5 µM) or ERK1/2 inhibitor (100 µM). Arrowheads: engulfed RBCs. Bars (A to D; F to I; L), colored symbols (J, K, M, N, P) or horizontal line (R) represent means of biological replicates, depicted as white objects for samples in (A-D, F-I, L) or grey/orange objects for phagosomes in (P and R). Error bars, ± S.E.M. Statistical comparisons between groups are unpaired Student's *t*-test (A to D; F to I; L) or ANOVA and Tukey's multiple comparisons (P, R). Data pooled from 3-5 (A to D; F to I) or 3 (P) independent experiments, or representative of 2 (E, K, Q, R) or 3 (J, L, M to O) independent experiments. Scale bar: 10 µm.

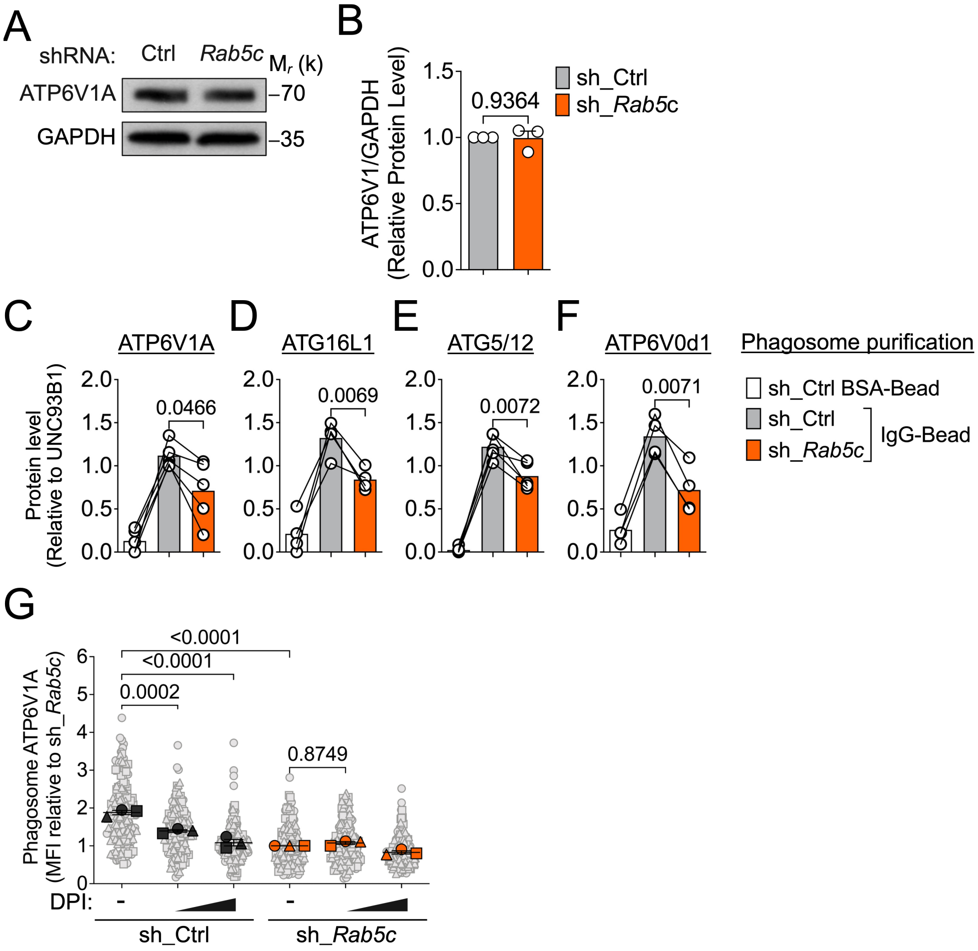


**Fig. S3.**

**Related to Figure 3.** RAW264.7 cells expressing sh_Ctrl or sh_*Rab5c*. (**A** and **B**) Immunoblot (A) and quantification (B) of ATP6V1A in the cell lysates. GAPDH, loading control. (**C** to **F**) Quantification of ATP6V1A (C), ATG16L1 (D), ATG5/12 (E), and ATP6V0d1 (F) on purified phagosomes. UNC93B1, loading control (*related to Fig. 3C*). (**G**) ATP6V1A MFI at phagosomes upon treatment with DPI (0.61 or 5 µM) prior to Op-zym. Bars (B to F) or colored symbols (G) represent means of biological replicates, each indicated as a gray (phagosome) or white (sample) object. Error bars, ± S.E.M. Statistical comparison between groups are unpaired Student′s *t*-test (B to F) or ANOVA and Tukey′s multiple comparisons (G). Data pooled from 3 (B, G) or 4-5 (C to F) independent experiments, or representative of 3 (A) independent experiments.

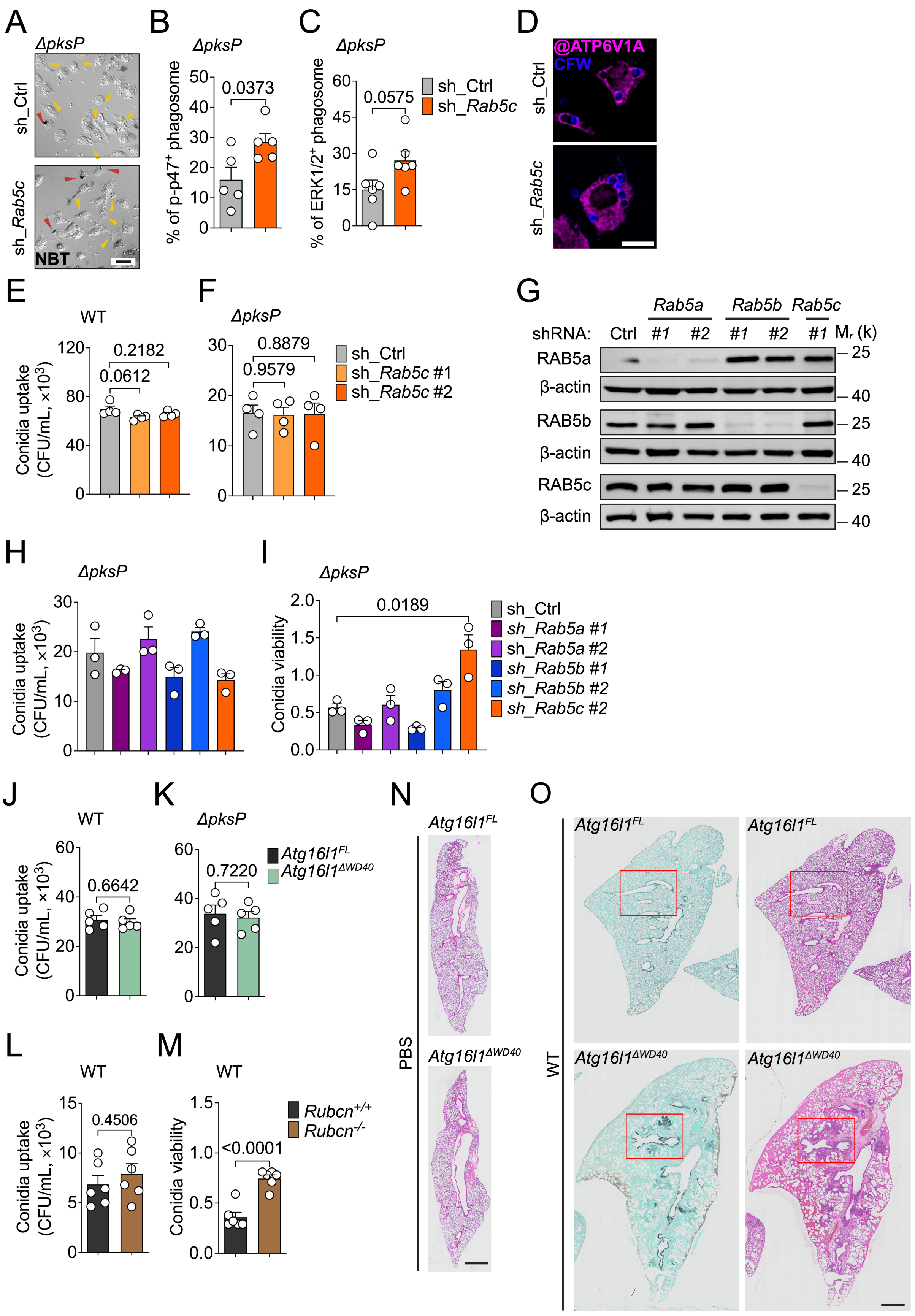

A
ΔpksP
sh_Ctrl
sh_Rab5c
NBT
B
ΔpksP
% of p-p47+ phagosome
0.0373
C
ΔpksP
% of ERK1/2+ phagosome
0.0575
sh_Ctrl
sh_Rab5c
D
@ATP6V1A
CFW
sh_Ctrl
sh_Rab5c
E
WT
Conidia uptake (CFU/mL, ×10³)
0.2182
0.0612
F
ΔpksP
0.8879
0.9579
sh_Ctrl
sh_Rab5c #1
sh_Rab5c #2
G
Rab5a
Rab5b
Rab5c
shRNA: Ctrl #1 #2 #1 #2 #1
Mr (k)
RAB5a
β-actin
RAB5b
β-actin
RAB5c
β-actin
H
ΔpksP
Conidia uptake (CFU/mL, ×10³)
I
ΔpksP
Conidia viability
0.0189
sh_Ctrl
sh_Rab5a #1
sh_Rab5a #2
sh_Rab5b #1
sh_Rab5b #2
sh_Rab5c #2
J
WT
Conidia uptake (CFU/mL, ×10³)
0.6642
K
ΔpksP
0.7220
Atg16l1FL
Atg16l1ΔWD40
L
WT
Conidia uptake (CFU/mL, ×10³)
0.4506
M
WT
Conidia viability
<0.0001
Rubcn+/+
Rubcn-/-
N
PBS
Atg16l1FL
Atg16l1ΔWD40
O
WT
Atg16l1FL
Atg16l1FL
Atg16l1ΔWD40
Atg16l1ΔWD40

**Fig. S4.**

**Related to Figure 4.** (**A** to **F**) BMDM expressing sh_Ctrl or sh_*Rab5c* were stimulated with wild-type (ATCC46645; WT) or Δ*pksP A. fumigatus* conidia. (**A**) DIC images of NBT assay. Arrowheads: $NBT^{+}$ (red) or $NBT^{-}$ (yellow) phagocytes. Scale bar: 20 μm. (**B** and **C**) Percentage of $p\text{-}p47^{phox}$- (B) and ERK1/2-decorated (C) phagosomes. (**D**) Confocal images of $ATP6V1A^{+}$ phagosomes. Scale bar: 10 μm. CFW: Calcofluor white. (**E** to **F**) Phagocytosis of WT (E) or Δ*pksP* (F) conidia. (**G** to **I**) BMDM expressing sh_Ctrl or indicated shRNAs (#1; #2) sequences were stimulated with Δ*pksP* conidia. Immunoblot of RAB5a, RAB5b, and RAB5c in the cell lysates (G). β-actin, loading control. Phagocytosis (H) and viability (I) of Δ*pksP* conidia. (**J** and **K**) Phagocytosis of WT conidia (J) or Δ*pksP* conidia (K) in $Atg16l1^{FL}$ and $Atg16l1^{\Delta WD40}$ BMDM. (**L** and **M**) Phagocytosis (L) and viability (M) of WT conidia in $Rubcn^{+/+}$ and $Rubcn^{-/-}$ BMDM. (**N**) Brightfield images of lung sections stained with H&E. Scale bar: 1,000 μm. (**O**) Brightfield images of lung sections stained with Grocott-Gomori′s methenamine silver (left) or H&E (right) (*related to Fig. 4O*). Scale bar: 1,000 μm. Bars (B, C, E, F, H, J to M) represent means of biological replicates (sample or mice), each indicated as a white object. Error bars, ± S.E.M. Statistical comparison between groups are unpaired Student′s *t*-test (B, C, E, F, J to M) or ANOVA and Tukey′s multiple comparisons (H and I). Data representative of 2 (A to D, G to I, K, N, O) or 3 (E, F, J, L, M) independent experiments.

**Movie S1.**

**Time-lapse spinning-disk confocal microscopy analysis of Op-zym phagocytosis in RAW264.7 cells expressing mCherry-RAB5c.** Image stacks were acquired every 20 s. Movie plays 5 frames/s; time, min:s. mCh-RAB5c fluorescence intensity is scaled using the mpl-Plasma LUT. Scale bar: 5 µm. *Related to Fig. 1A*. Type or paste caption here.

**Movie S2.**

**Time-lapse spinning-disk confocal microscopy analysis of Op-zym phagocytosis in RAW264.7 cells expressing mCherry-LC3B and transduced with sh_Ctrl or sh_*Rab5c*.** Image stacks were acquired every 30 s. Movie plays 3 frames/s; time, min:s. Scale bar: 5 µm. *Related to Fig. S1D*.

**Movie S3.**

**Time-lapse spinning-disk confocal microscopy analysis of Op-zym phagocytosis in RAW264.7 cells expressing p40$^{phox}$-PX-Venus and transduced with sh_Ctrl or sh_*Rab5c*.** Image stacks were acquired every 30 s. Movie plays 2 frames/s; time, min:s. mCh-RAB5c fluorescence intensity is scaled using the SMART LUT. Scale bar: 10 µm. *Related to Fig. 2C.*

**Movie S4.**

**Time-lapse spinning-disk confocal microscopy analysis of Op-zym phagocytosis in RAW264.7 cells expressing ATP6V0a3-EGFP and transduced with sh_Ctrl or sh_*Rab5c*.** Image stacks were acquired every 30 s. Movie plays 2 frames/s; time, min:s. Scale bar: 5 µm. *Related to Fig. 3D.*
